# Human subcortical brain asymmetries in 15,847 people worldwide reveal effects of age and sex

**DOI:** 10.1007/s11682-016-9629-z

**Published:** 2016-10-13

**Authors:** Tulio Guadalupe, Samuel R. Mathias, Theo G. M. vanErp, Christopher D. Whelan, Marcel P. Zwiers, Yoshinari Abe, Lucija Abramovic, Ingrid Agartz, Ole A. Andreassen, Alejandro Arias-Vásquez, Benjamin S. Aribisala, Nicola J. Armstrong, Volker Arolt, Eric Artiges, Rosa Ayesa-Arriola, Vatche G. Baboyan, Tobias Banaschewski, Gareth Barker, Mark E. Bastin, Bernhard T. Baune, John Blangero, Arun L.W. Bokde, Premika S.W. Boedhoe, Anushree Bose, Silvia Brem, Henry Brodaty, Uli Bromberg, Samantha Brooks, Christian Büchel, Jan Buitelaar, Vince D. Calhoun, Dara M. Cannon, Anna Cattrell, Yuqi Cheng, Patricia J. Conrod, Annette Conzelmann, Aiden Corvin, Benedicto Crespo-Facorro, Fabrice Crivello, Udo Dannlowski, Greig I. de Zubicaray, Sonja M.C. de Zwarte, Ian J. Deary, Sylvane Desrivières, Nhat Trung Doan, Gary Donohoe, Erlend S. Dørum, Stefan Ehrlich, Thomas Espeseth, Guillén Fernández, Herta Flor, Jean-Paul Fouche, Vincent Frouin, Masaki Fukunaga, Jürgen Gallinat, Hugh Garavan, Michael Gill, Andrea Gonzalez Suarez, Penny Gowland, Hans J. Grabe, Dominik Grotegerd, Oliver Gruber, Saskia Hagenaars, Ryota Hashimoto, Tobias U. Hauser, Andreas Heinz, Derrek P. Hibar, Pieter J. Hoekstra, Martine Hoogman, Fleur M. Howells, Hao Hu, Hilleke E. Hulshoff Pol, Chaim Huyser, Bernd Ittermann, Neda Jahanshad, Erik G. Jönsson, Sarah Jurk, Rene S. Kahn, Sinead Kelly, Bernd Kraemer, Harald Kugel, Jun Soo Kwon, Herve Lemaitre, Klaus-Peter Lesch, Christine Lochner, Michelle Luciano, Andre F. Marquand, Nicholas G. Martin, Ignacio Martínez-Zalacaín, Jean-Luc Martinot, David Mataix-Cols, Karen Mather, Colm McDonald, Katie L. McMahon, Sarah E. Medland, José M. Menchón, Derek W. Morris, Omar Mothersill, Susana Munoz Maniega, Benson Mwangi, Takashi Nakamae, Tomohiro Nakao, Janardhanan C. Narayanaswaamy, Frauke Nees, Jan E. Nordvik, A. Marten H. Onnink, Nils Opel, Roel Ophoff, Marie-Laure Paillère Martinot, Dimitri Papadopoulos Orfanos, Paul Pauli, Tomáš Paus, Luise Poustka, Janardhan YC. Reddy, Miguel E. Renteria, Roberto Roiz-Santiáñez, Annerine Roos, Natalie A. Royle, Perminder Sachdev, Pascual Sánchez-Juan, Lianne Schmaal, Gunter Schumann, Elena Shumskaya, Michael N. Smolka, Jair C. Soares, Carles Soriano-Mas, Dan J. Stein, Lachlan T. Strike, Roberto Toro, Jessica A. Turner, Nathalie Tzourio-Mazoyer, Anne Uhlmann, Maria Valdés Hernández, Odile A. van den Heuvel, Dennis van der Meer, Neeltje E.M . van Haren, Dick J. Veltman, Ganesan Venkatasubramanian, Nora C. Vetter, Daniella Vuletic, Susanne Walitza, Henrik Walter, Esther Walton, Zhen Wang, Joanna Wardlaw, Wei Wen, Lars T. Westlye, Robert Whelan, Katharina Wittfeld, Thomas Wolfers, Margaret J. Wright, Jian Xu, Xiufeng Xu, Je-Yeon Yun, JingJing Zhao, Barbara Franke, Paul M. Thompson, David C. Glahn, Bernard Mazoyer, Simon E. Fisher, Clyde Francks

**Affiliations:** 1 Language & Genetics Department, Max Planck Institute for Psycholinguistics, Nijmegen, The Netherlands; 2 International Max Planck Research School for Language Sciences, Nijmegen, The Netherlands; 3 Department of Psychiatry, Yale School of Medicine, New Haven, CT 06519 USA; 4 Department of Psychiatry and Human Behavior, University of California, Irvine, CA USA; 5 Imaging Genetics Center, Institute for Neuroimaging & Informatics, Keck School of Medicine of the University of Southern California, Marina del Rey, CA USA; 6 Molecular and Cellular Therapeutics, The Royal College of Surgeons, Dublin 2, Ireland; 7 Donders Centre for Cognitive Neuroimaging, Donders Institute for Brain, Cognition and Behaviour, Radboud University, Nijmegen, The Netherlands; 8 Department of Psychiatry, Graduate School of Medical Science, Kyoto Prefectural University of Medicine, Kyoto, Japan; 9 Brain Centre Rudolf Magnus, University Medical Centre Utrecht, Utrecht, The Netherlands; 10 NORMENT - KG Jebsen Centre, Institute of Clinical Medicine, University of Oslo, Oslo, Norway; 11 Department of Research and Development, Diakonhjemmet Hospital, Oslo, Norway; 12 Department of Clinical Neuroscience, Psychiatry Section, Karolinska Institutet, Stockholm, Sweden; 13 NORMENT - KG Jebsen Centre, Division of Mental Health and Addiction, Oslo University Hospital, Oslo, Norway; 14 Department of Human Genetics, Donders Institute for Brain, Cognition and Behaviour, Radboud university medical center, Nijmegen, The Netherlands; 15 Department of Psychiatry, Donders Institute for Brain, Cognition and Behaviour, Radboud University Medical Center, Nijmegen, The Netherlands; 16 Department of Cognitive Neuroscience, Radboud University Medical Center, Nijmegen, The Netherlands; 17 Department of Computer Science, Lagos State University, Lagos, Nigeria; 18 Brain Research Imaging Centre, University of Edinburgh, Edinburgh, UK; 19 Centre for Healthy Brain Ageing, School of Psychiatry, University of New South Wales (UNSW), Sydney, Australia; 20 Mathematics and Statistics, Murdoch University, Murdoch, Australia; 21 Department of Psychiatry, University of Münster, Münster, Germany; 22 Institut National de la Santé et de la Recherche Médicale, INSERM Unit 1000 “Neuroimaging & Psychiatry”, University Paris Sud, University Paris Descartes -Sorbonne Paris Cité, Paris, France; 23 Department of Psychiatry, University Hospital Marqués de Valdecilla, School of Medicine, University of Cantabria-IDIVAL, Santander, Spain; 24 CIBERSAM, Centro Investigación Biomédica en Red Salud Mental, Santander, Spain; 25 Imaging Genetics Center, Institute for Neuroimaging & Informatics, Keck School of Medicine of the University of Southern California, Los Angeles, USA; 26 Department of Child and Adolescent Psychiatry and Psychotherapy, Central Institute of Mental Health, Medical Faculty Mannheim, Heidelberg University, Square J5, 68159 Mannheim, Germany; 27 Centre for Neuroimaging Sciences, Institute of Psychiatry, Psychology & Neuroscience, King’s College London, London, UK; 28 Centre for Cognitive Ageing and Cognitive Epidemiology, Psychology, University of Edinburgh, Edinburgh, UK; 29 Centre for Clinical Brain Sciences, University of Edinburgh, Edinburgh, UK; 30 Scottish Imaging Network, A Platform for Scientific Excellence (SINAPSE) Collaboration, Department of Neuroimaging Sciences, University of Edinburgh, Edinburgh, UK; 31 Discipline of Psychiatry, School of Medicine, University of Adelaide, Adelaide, SA 5005 Australia; 32 Department of Genetics, Texas Biomedical Research Institute, San Antonio, TX USA; 33 South Texas Diabetes and Obesity Institute, University of Texas Rio Grande Valley School of Medicine, Brownsville, TX USA; 34 Discipline of Psychiatry, School of Medicine and Trinity College Institute of Neurosciences, Trinity College Dublin, Dublin, Ireland; 35 Department of Psychiatry, VU University Medical Center, Amsterdam, The Netherlands; 36 Department of Anatomy & Neurosciences, VU University Medical Center, Amsterdam, The Netherlands; 37 Neuroscience Campus Amsterdam, VU/VUMC, Amsterdam, The Netherlands; 38 Department of Psychiatry, National Institute of Mental Health and Neurosciences, Bangalore, India; 39 University Clinic for and Adolescent Psychiatry UCCAP, University of Zurich, Zurich, Switzerland; 40 Neuroscience Center Zurich, University of Zurich and ETH Zurich, Zurich, Switzerland; 41 Centre for Healthy Brain Ageing (CHeBA), & Dementia Collaborative Research Centre, School of Psychiatry, UNSW Medicine, University of New South Wales, Sydney, Australia; 42 University Medical Centre Hamburg-Eppendorf, House W34, 3.OG, Martinistr. 52, 20246 Hamburg, Germany; 43 Department of Psychiatry, University of Cape Town, Cape Town, South Africa; 44 Donders Institute for Brain, Cognition and Behaviour, Raboud University, Nijmegen, The Netherlands; 45 Karakter Child and Adolescent Psychiatry, Radboud university medical center, Nijmegen, The Netherlands; 46 Departments of Electrical and Computer Engineering,Neurosciences, Computer Science, and Psychiatry, The University of New Mexico, Albuquerque, NM USA; 47 The Mind Research Network, Albuquerque, NM USA; 48 Centre for Neuroimaging, Cognition & Genomics (NICOG), Clinical Neuroimaging Laboratory, NCBES Galway Neuroscience Centre, College of Medicine, Nursing and Health Sciences, National University of Ireland Galway, Galway, H91 TK33, Ireland; 49 Medical Research Council - Social, Genetic and Developmental Psychiatry Centre, Institute of Psychiatry, Psychology & Neuroscience, King’s College London, London, UK; 50 Department of Psychiatry, The First Affiliated Hospital of Kunming Medical University, Kunming, China; 51 Department of Psychiatry, Universite de Montreal, CHU Ste Justine Hospital, Montréal, Canada; 52 Department of Psychological Medicine and Psychiatry, Institute of Psychiatry, Psychology & Neuroscience, King’s College London, London, UK; 53 Department of Psychology (Biological Psychology, Clinical Psychology, and Psychotherapy), University of Würzburg, Germany, Tübingen, Würzburg, Germany; 54 Department of Child and Adolescent Psychiatry and Psychotherapy, University of Tübingen, Tübingen, Germany; 55 Department of Psychiatry, Trinity College Dublin, Dublin, Ireland; 56 UMR5296 CNRS, CEA and University of Bordeaux, Bordeaux, France; 57 Department of Psychiatry, University of Münster, Münster, Germany; 58 Department of Psychiatry, University of Marburg, Marburg, Germany; 59 Faculty of Health and Institute of Health and Biomedical Innovation, Queensland University of Technology (QUT), Brisbane City, Australia; 60 Cognitive Genetics and Cognitive Therapy Group, Neuroimaging, Cognition & Genomics Centre (NICOG), School of Psychology and Discipline of Biochemistry, National University of Ireland Galway, SW4 794, Galway, Ireland; 61 Department of Psychiatry & trinity College Institute of Neuroscience, Trinity College Dublin, Dublin, Ireland; 62 Sunnaas Rehabilitation Hospital HT, Nesodden, Norway; 63 Department of Psychology, University of Oslo, Oslo, Norway; 64 Department of Child and Adolescent Psychiatry, Faculty of Medicine of the TU Dresden, Dresden, Germany; 65 Department of Psychiatry, Massachusetts General Hospital, Boston, USA; 66 Martinos Center for Biomedical Imaging, Massachusetts General Hospital, Charlestown, USA; 67 NORMENT - KG Jebsen Centre, Department of Psychology, University of Oslo, Oslo, Norway; 68 Department of Cognitive and Clinical Neuroscience, Central Institute of Mental Health, Medical Faculty Mannheim, Heidelberg University, Square J5, Mannheim, Germany; 69 Department of Psychiatry and Mental Health, University of Cape Town, Cape Town, South Africa; 70 Neurospin, Commissariat à l’Energie Atomique, CEA-Saclay Center, Paris, France; 71 Division of Cerebral Integration, National Institute for Physiological Sciences, Okazaki, Japan; 72 Department of Psychiatry and Psychotherapy, University Medical Center Hamburg-Eppendorf (UKE), Martinistrasse 52, 20246 Hamburg, Germany; 73 Departments of Psychiatry and Psychology, University of Vermont, Burlington, VT 05405 USA; 74 Trinity College Institute of Neuroscience, Trinity College, Dublin, Ireland; 75 Service of Neurology, University Hospital Marqués de Valdecilla (IDIVAL), University of Cantabria (UC), Santander, Spain; 76 CIBERNED, Centro de Investigación Biomédica en red Enfermedades Neurodegenerativas, Madrid, Spain; 77 Sir Peter Mansfield Imaging Centre School of Physics and Astronomy, University of Nottingham, University Park, Nottingham, UK; 78 Department of Psychiatry, University Medicine Greifswald, Greifswald, Germany; 79 Department of Psychiatry and Psychotherapy, HELIOS Hospital Stralsund, Stralsund, Germany; 80 Department of Psychiatry, University of Münster, Münster, Germany; 81 Center for Translational Research in Systems Neuroscience and Psychiatry, Department of Psychiatry and Psychotherapy, University Medical Center, D-37075 Göttingen, Germany; 82 Centre for Cognitive Ageing and Cognitive Epidemiology, University of Edinburgh, Edinburgh, UK; 83 Molecular Research Center for Children’s Mental Development, United Graduate School of Child Development, Osaka University, Osaka, Japan; 84 Department of Psychiatry, Osaka University Graduate School of Medicine, Osaka, Japan; 85 University Clinic for Child and Adolescent Psychiatry (UCCAP), University of Zurich, Zurich, Switzerland; 86 Wellcome Trust Centre for Neuroimaging, University College London, London, UK; 87 UCL Max Planck Centre for Computational Psychiatry and Ageing, University College London, London, UK; 88 Department of Psychiatry and Psychotherapy, Campus Charité Mitte, Charité, Universitätsmedizin Berlin, Charitéplatz 1, Berlin, Germany; 89 Department of Psychiatry, University of Groningen, University Medical Center Groningen, Groningen, The Netherlands; 90 Shanghai Mental Health Center, Shanghai Jiao Tong University School of Medicine, No. 600 Wan Ping Nan Road, Shanghai, 200030 China; 91 De Bascule, Academic Center for Child and Adolescent Psychiatry, Amsterdam, The Netherlands; 92 AMC, department of child and adolescent psychiatry, Amsterdam, The Netherlands; 93 Physikalisch-Technische Bundesanstalt (PTB), Braunschweig and Berlin, Germany; 94 NORMENT, KG Jebsen Centre for Psychosis Research, Institute of Clinical Medicine. Psychiatry section, University of Oslo, Oslo, Norway; 95 Department of Psychiatry and Neuroimaging Center, Technische Universität Dresden, Dresden, Germany; 96 Imaging Genetics Center, Institute for Neuroimaging & Informatics, Keck School of Medicine of the University of Southern California, Los Angeles, 90292 USA; 97 Department of Clinical Radiology, University of Münster, Münster, Germany; 98 Department of Psychiatry & Behavioral Science, Seoul National University College of Medicine, Seoul, Republic of Korea; 99 Institute of Human Behavioral Medicine, SNU-MRC, Seoul, Republic of Korea; 100 Department of Brain & Cognitive Sciences, College of Natural Science, Seoul National University, Seoul, Republic of Korea; 101 Division of Molecular Psychiatry, Center of Mental Health, University of Würzburg, Würzburg, Germany; 102 Department of Translational Neuroscience, School for Mental Health and Neuroscience (MHeNS), Maastricht University, Maastricht, The Netherlands; 103 Department of Psychiatry, University of Stellenbosch and MRC Unit on Anxiety & Stress Disorders, Tygerberg, Cape Town South Africa; 104 Department of Neuroimaging, Centre for Neuroimaging Sciences, Institute of Psychiatry, King’s College London, London, UK; 105 QIMR Berghofer Medical Research Institute, Brisbane, Australia; 106 Department of Psychiatry, Bellvitge University Hospital - Institut d’Investigació Biomèdica de Bellvitge (IDIBELL), Barcelona, Spain; 107 Institut National de la Santé et de la Recherche Médicale, INSERM Unit 1000 “Neuroimaging & Psychiatry”, University Paris Sud, University Paris Descartes - Sorbonne Paris Cité, and Maison de Solenn, Paris, France; 108 Maison de Solenn, Paris, France; 109 Department of Clinical Neuroscience,Centre for Psychiatric Research and Education, Karolinska Institutet, Stockholm, Sweden; 110 Centre for Advanced Imaging, University of Queensland, Brisbane, Australia; 111 CIBER Salud Mental (CIBERSAM), Instituto de Salud Carlos III, Barcelona, Spain; 112 Department of Clinical Sciences, University of Barcelona, Barcelona, Spain; 113 UT Center of Excellence on Mood Disorders, Department of Psychiatry and Behavioral Sciences, UT Houston Medical School, Houston, TX USA; 114 Department of Neural Computation for Decision-Making, ATR Brain Information Communication Research Laboratory Group, Kyoto, Japan; 115 Department of Neuropsychiatry, Graduate School of Medical Science, Kyushu University, Fukuoka, Japan; 116 Center for Neurobehavioral Genetics, University of California, Los Angeles, USA; 117 AP-HP, Department of Adolescent Psychopathology and Medicine, Maison de Solenn, Cochin Hospital, Paris, France; 118 Department of Psychiatry and Psychotherapy, University of Würzburg, Würzburg, Germany; 119 Rotman Research Institute, Baycrest and Departments of Psychology and Psychiatry, University of Toronto, M6A 2E1, Toronto, ON Canada; 120 Department of Child and Adolescent Psychiatry and Psychotherapy, Medical University of Vienna, Vienna, Austria; 121 Department of Psychiatry, Neuroscience Campus Amsterdam, VU University Medical Center, Amsterdam, The Netherlands; 122 Department of Psychiatry and Behavioral Sciences, University of Texas Health Science Center at Houston, Houston, TX 77054 USA; 123 Department of Psychobiology and Methodology of Health Sciences, Universitat Autònoma de Barcelona, Barcelona, Spain; 124 Department of Psychiatry, University of Cape Town and MRC Unit on Anxiety & Stress Disorders, Cape Town, South Africa; 125 Queensland Brain Institute, The University of Queensland, Brisbane, Australia; 126 Laboratory of Human Genetics and Cognitive Functions, Institut Pasteur, 75015 Paris, France; 127 Department of Psychology, Georgia State University, Atlanta, GA USA; 128 Department of Neuroscience, Georgia State University, Atlanta, GA USA; 129 Department of Psychiatry and Mental Health, University of Cape Town, Observatory, Cape Town, South Africa; 130 Department of Psychiatry, VU University Medical Center, Amsterdam, The Netherlands; 131 Neuroscience Center Zurich, University of Zurich and ETH Zurich, Zurich, Switzerland; 132 Zurich Center for Integrative Human Physiology, University of Zurich, Zurich, Switzerland; 133 Department of Psychology, University College Dublin, Dublin, Ireland; 134 German Center for Neurodegenerative Diseases (DZNE), Site Rostock, Greifswald, Germany; 135 Queensland Brain Institute and Centre for Advanced Imaging, The University of Queensland, Brisbane, Australia; 136 Seoul National University Hospital, Seoul, Republic of Korea; 137 Cognitive Genetics and Therapy Group, School of Psychology & Discipline of Biochemistry, National University of Ireland Galway, Galway, SW4 794 Ireland; 138 Department of Psychiatry, Yale University, New Haven, CT 06511 USA; 139 Olin Neuropsychiatric Research Center, Hartford, CT 06114 USA; 140 School of Psychology, Shaanxi Normal University, Xi’an, China

**Keywords:** Subcortical brain asymmetry, Age, Handedness, Sex, Enigma, Heritability, Meta-analysis

## Abstract

The two hemispheres of the human brain differ functionally and structurally. Despite over a century of research, the extent to which brain asymmetry is influenced by sex, handedness, age, and genetic factors is still controversial. Here we present the largest ever analysis of subcortical brain asymmetries, in a harmonized multi-site study using meta-analysis methods. Volumetric asymmetry of seven subcortical structures was assessed in 15,847 MRI scans from 52 datasets worldwide. There were sex differences in the asymmetry of the globus pallidus and putamen. Heritability estimates, derived from 1170 subjects belonging to 71 extended pedigrees, revealed that additive genetic factors influenced the asymmetry of these two structures and that of the hippocampus and thalamus. Handedness had no detectable effect on subcortical asymmetries, even in this unprecedented sample size, but the asymmetry of the putamen varied with age. Genetic drivers of asymmetry in the hippocampus, thalamus and basal ganglia may affect variability in human cognition, including susceptibility to psychiatric disorders.

**Electronic supplementary material:**

The online version of this article (doi:10.1007/s11682-016-9629-z) contains supplementary material, which is available to authorized users.

## Introduction

Left–right differentiation of the central nervous system (CNS) results in anatomical, functional, and behavioral asymmetries in many organisms (Ocklenburg and Gunturkun 2012). Humans are no exception: functions including language, visuospatial cognition, and hand–motor control are asymmetrically organized between hemispheres in a typical human brain (Haaland and Harrington 1996; Mellet et al. 2014). At the population level, these asymmetries show clear directional biases, or *lateralizations* (Bryden 1982). Handedness is the most overt example: around 90 % of people have a right-hand preference, a strong bias not seen in other species including our closest evolutionary relatives, the apes (Hopkins et al. 2011).

Functional and structural lateralization of the human brain may be influenced by left-right differences in gene expression (Francks 2015), as recently demonstrated in language-related regions of the adult superior temporal cortex (Karlebach and Francks 2015). Even so, lateralization varies markedly across individuals. Women and men show average differences in asymmetry, as well. Men show, on average, more pronounced asymmetries in superior temporal language regions of the cerebral cortex than women, based on brain magnetic resonance imaging (MRI) data from over 3000 people (Guadalupe et al. 2015). Genes involved in steroid hormone biology may affect the degree of lateralization in both men and women (Guadalupe et al. 2015). Another trait linked to cerebral lateralization is handedness (Willems et al. 2014): the largest study of cerebral cortical structural differences by handedness showed weak associations with changes in surface area of the left precentral sulcus (Guadalupe et al. 2014a), consistent with prior reports (Amunts et al. 1996; Foundas et al. 1998). Left-handers have a slightly higher incidence of atypical functional hemispheric language dominance (Mazoyer et al. 2014). Alterations of cerebral cortical lateralization have also been linked to cognitive and psychiatric disorders , including language-related impairments (Altarelli et al. 2014; Herbert et al. 2005), autism (Eyler et al. 2012; Herbert et al. 2005), schizophrenia (SCZ; Oertel-Knochel et al. 2012), and substance-use disorders (Balconi and Finocchiaro 2015).

In contrast to the cerebral cortex, lateralizations of human subcortical structures and the hippocampus have not been well studied, nor the factors that might affect their individual differences or roles in lateralized cognition. Most investigations have been in clinical contexts, where differences between cases and controls in asymmetry patterns of subcortical structures have been linked to various neuropsychiatric disorders. For example, abnormal asymmetries in the basal ganglia, particularly of the globus pallidus and caudate nucleus, have been observed in cases of attention-deficit/hyperactivity disorder (ADHD; Hynd et al. 1993), and in developmental stuttering and Tourette’s syndrome (TS; Foundas et al. 2013; Singer et al. 1993). Abnormal asymmetry of the striatum has been linked to prenatal alcohol or methamphetamine exposure (Roos et al. 2014; Willford et al. 2010). Changes in thalamic asymmetry have been found in cases of TS (Lee et al. 2006) and SCZ (Zhou et al. 2003). Regarding limbic system structures, studies of major depression (Xia et al. 2004), obsessive-compulsive disorder (Szeszko et al. 1999), SCZ (Niemann et al. 2000), anorexia nervosa (Titova et al. 2013), and age-related memory impairment (Soininen et al. 1994) have shown abnormal asymmetries of the hippocampus, which in patients with temporal lobe epilepsy also included the amygdala (Cendes et al. 1993). Abnormal asymmetries of the amygdala have also been reported in SCZ (Niu et al. 2004) and in cocaine addiction (Makris et al. 2004). Some of these disorders differ in their prevalence between sexes and by handedness (Castellanos et al. 2001; DeLisi et al. 2002; Niemann et al. 2000). Interestingly, sex differences in subcortical asymmetries have been suggested to have an etiological role in TS (Zimmerman et al. 2000) and SCZ (Niu et al. 2004). These findings suggest that, in addition to the more salient cerebral cortical asymmetries, asymmetries of the subcortical nuclei also play a role in brain health and disease.

Despite these intriguing initial findings with respect to disease states, decades of research have failed to answer definitively how brain asymmetries in the healthy population are linked to basic biological factors such as age, sex, and handedness. This is partly because many brain asymmetries and their normal variability are subtle, and difficult to measure reliably in small studies (tens to low hundreds of subjects are typical). Regarding sexual dimorphisms, a sex difference in asymmetry of the amygdala has been reported (Niu et al. 2004), while no sex difference was detected in another study (Szabo et al. 2001). For striatal asymmetry, no significant sex differences were observed by three studies (Abedelahi et al. 2013; Giedd et al. 1996; Wyciszkiewicz and Pawlak 2014), although a sex difference in putamen asymmetry was suggested to affect TS etiology (Zimmerman et al. 2000). Sexual dimorphism in thalamic asymmetry has been recently reported (Kang et al. 2015) but not replicated. Asymmetry of striatal nuclei changes with age (Abedelahi et al. 2013; Yamashita et al. 2011), but prior studies of subcortical structures have tended to look at age and asymmetry as separate aspects of study (Caviness et al. 1996; Giedd et al. 1996). Left-handedness has not been robustly investigated in relation to subcortical asymmetries, as there are so few left-handers in most datasets (Foundas et al. 1998; Kloppel et al. 2007). Likewise, in clinical studies, possible effects of sex, age, and handedness have not often been investigated, either as a result of restricted inclusion criteria, or otherwise not considering these factors in their analyses (e.g. Kang et al. 2015; Yamashita et al. 2011).

The present study was the first by the Lateralization working-group embedded within the ENIGMA (Enhancing Imaging Genetics through Meta-Analysis) Consortium (Thompson et al. 2014). Our goal was to detect effects of sex, handedness, and age on the normal variability in subcortical asymmetries, through a harmonized multi-site study using meta-analysis methods, based on 52 healthy control and population-based datasets which comprised a total of 15,847 participants. All brain magnetic resonance (MR) images were analyzed using a single, uniform protocol, despite inevitable heterogeneity in image acquisition (Hibar et al. 2015; Stein et al. 2012). This study was by two orders of magnitude the largest ever of asymmetry with respect to subcortical structures of the human brain, and factors affecting its variability. This allowed us to establish subtle but definitive findings of sex and age-related effects on some of the structures, where previously the literature has been inconsistent and contradictory (see Discussion). We also measured the heritabilities of subcortical and hippocampal asymmetries in a large family dataset, as previous studies have suggested these to be partially heritable (Eyler et al. 2014; Hulshoff Pol et al. 2006; Renteria 2013). This heritability screen is a valuable precursor to future genome-wide association studies of laterality in brain traits, as well as identifying genetic overlap between asymmetries and cognitive or psychiatric disorders.

## Methods

### Datasets

The participating sites were members of the Lateralization working-group within the ENIGMA Consortium (Thompson et al. 2014), who contributed data from 52 independent samples to this study comprising a total of 15,847 healthy participants (7524 males and 8323 females). Samples were drawn from the general population or comprised healthy controls from clinical studies. Table 1 and Supplemental Information S1 summarize the datasets’ sample sizes and age distributions. Each dataset and its image acquisition protocols are described in Supplemental Information S2.

**Table 1:** List of contributing datasets (arranged alphabetically in two columns), their sample sizes split by sex, and their median ages. Each dataset is also given a suffix number code for reference to Fig. 2, Fig. 3, and Supplemental Information S5

Dataset	N		Median age (years)	Dataset	N		Median age (years)
	Males	Females			Males	Females	
BIG 1.5 T_1_	733	728	23	OCD-Kunming 3 T_27_	27	68	25
BIG 3T_2_	579	729	22	OCD-Kyoto 1.5 T_28_	25	23	30
BIL & GIN_3_	221	232	24	OCD-Kyoto 3 T_29_	20	22	30
BP-Houston_4_	79	94	19	OCD-London_30_	12	21	32
CIAM_5_	16	14	27	OCD-Shangai_31_	21	17	25
CLiNG_6_	132	191	24	OCD-SNU A_32_	53	26	25
FBIRN_7_	129	54	37	OCD-SNU B_33_	97	59	24
HMS_8_	21	34	41	OCD-SNU C_34_	115	72	24
HUBIN_9_	69	33	46	OCD-SU_35_	11	18	29
IMAGEN_10_	735	847	15	OCD-VUmc Amsterdam 1.5 T_36_	16	38	34
IMpACT_11_	61	80	32	OCD-VUmc Amsterdam 3 T_37_	20	22	38
LBC-1936_12_	282	274	73	OCD-Zürich_38_	15	23	17
MAS_13_	224	280	78	Osaka 1.5 T_39_	206	231	33
MCIC_14_	103	60	28	Osaka 3 T_40_	131	106	24
Meth-CT_15_	50	13	25	PAFIP-IDIVAL1_41_	51	30	26
MüNC_16_	327	420	32	PAFIP-IDIVAL2_42_	69	45	29
NCNG_17_	105	222	54	PAFIP-IDIVAL3_43_	13	21	69
NESDA_18_	23	43	41	QTIM_44_	169	422	22
NeuroIMAGE_19_	180	208	17	SHIP-2_45_	538	572	56
OATS_20_	87	153	69	SHIP-Trend_46_	994	1046	52
OCD-AMC_21_	9	18	14	STROKEMRI_47_	19	33	45
OCD-Barcelona_22_	30	36	33	TCD|NUIG_48_	116	145	28
OCD-Fukuoka_23_	16	25	37	TOP_49_	159	144	34
OCD-India 1.5T_24_	34	12	26	UCLA|NL BP_50_	82	84	46
OCD-India 3T_25_	95	60	26	UMCU_51_	166	121	29
OCD-Kunming 1.5T_26_	13	27	31	Würzburg|Tübingen_52_	24	29	44

Handedness of participants was known for a subset of the overall sample. The method of assessment varied per dataset (see Supplemental Information S3). An ambidextrous category was not included and only datasets with enough left-handers to perform statistical comparisons were considered. In total, 959 and 11,236 subjects were left- and right-handed, respectively.

The final numbers of subjects and datasets that were used for meta-analyses differed per test and structure according to the availability of covariate and structure-specific volumetric information, and the minimum sample-size criteria. Details are given below per analysis.

### Image acquisition and subcortical segmentation

Image acquisition and subcortical volume measurement has been described in previous reports from the ENIGMA Consortium (e.g. (Hibar et al. 2015; Stein et al. 2012) , and is consistent enough to detect SNP effects at a genome-wide significant level, which individually account for less than 1 % of the variance in structure volumes. To summarize, T1-weighted brain structural MRI scans were acquired at multiple different sites using scanners of mostly 1.5 or 3 Tesla field strengths. One dataset (QTIM) was acquired with a 4 Tesla field strength scanner. See Supplemental Information S2 for detailed information on manufacturers and image acquisition parameters per dataset. All sites followed the same protocol for segmentation of subcortical structures, volume computation, and quality control. The protocol is downloadable from the ENIGMA website (http://enigma.ini.usc.edu/protocols/imaging-protocols/). Specifically, image pre-processing and subcortical segmentation were done with FreeSurfer versions 4.3 through to 5.3 (Fischl et al. 2002), using the “-recon-all” pipeline and default settings. This pipeline performs automated bias field correction, spatial normalization, skull stripping, and segments brain tissue into cortical gray/white matter, as well as into several non-cortical tissues. This resulted in volume estimates for the following seven bilaterally paired structures: nucleus accumbens, amygdala, caudate nucleus, globus pallidus, hippocampus, putamen, and thalamus, and estimates of total intracranial volume (ICV).

Quality control was performed separately by each of the contributing sites, and followed the harmonized protocol developed by the ENIGMA consortium (http://enigma.ini.usc.edu/protocols/imaging-protocols/). The protocol consisted of visually checking individual images, plotted from a set of axial slices. Volume estimates derived from poorly segmented structures (i.e. where tissue labels were assigned incorrectly) were excluded from each site’s datasets and subsequent analyses. In addition, a number of checks were performed to assess potential errors in the left-right orientation of the data (see Supplemental Information S4 for details).

### Within-dataset analyses

For each dataset and each of the seven bilaterally paired structures, the volumetric asymmetries, descriptive and statistical analyses were computed at each participating site using a single script in R (R Development Core Team; 2012), on table-formatted data. Asymmetry Indices (AI) were defined as the relative volume difference between the left and right structure in relation to its total bilateral volume: (Left - Right)/(Left + Right). To exclude possible outliers in volumes or AIs we used an adaptive SD threshold (SD_Thresh_) depending on each dataset’s sample size (*N* < 150 ⇒ SD_Thresh_ = 2.5; 150 ≥ *N* ≥ 1000 ⇒ SD_Thresh_ = 3; *N* > 1000 ⇒ SD_Thresh_ = 3.5). Statistical tests were run on the seven subcortical AIs separately. Differences between sexes or handedness groups were assessed by Welch’s two-sample t-test, to avoid assuming balanced group sizes and equal variances (Ruxton 2006). Tests were performed on residualised AIs, after removing effects of age and ICV (and sex for the handedness tests) by linear regression. Possible non-linear effects of ICV were investigated using the BIG sample but found to be negligible (Supplemental Information S5), hence all analyses were performed using only linear correction for this covariate. The effects of age on AIs were estimated by ANCOVAs, modelled together with sex and ICV as covariate factors.

This approach supported the subsequent application of meta-analysis methodology, through using within-site summary statistics, and without sites needing to share primary data.

### AI heterogeneity between datasets

For each of the seven AIs we assessed heterogeneity due to dataset differences through analyses of variance, with ‘dataset’ and ‘FreeSurfer version’ as the main factors. For this analysis we re-computed the total AI variance for a given structure and partitioned it into between-and within-'dataset’ contributions, and between- and within-'FreeSurfer version’ contributions. This allowed us to calculate estimates of eta-squared (η^2^), i.e., the percentage of the total variance explained by each factor. Given that individual sites ran their analyses on one version of FreeSurfer only, we computed main effects but not their potential interactions.

### Meta-analyses

We combined the test statistics obtained across datasets by means of random-effect meta-analyses (Borenstein et al. 2010). This method calculates and tests the significance of a pooled effect while weighting each dataset’s contribution to the overall effect by the inverse of its error variance. In contrast to a fixed-effect meta-analysis test, this method also takes into account the amount of variability present between effects from different studies in its calculation, and hence does not make strong assumptions regarding equal effects underlying all datasets (Borenstein et al. 2010).

For meta-analyses of sex and handedness effects, we used the mean group differences in residualised AIs and recomputed the standard errors from 95 % confidence intervals and degrees of freedom generated by the Welch’s two sample t-tests. For meta-analyses of age-effects, we used the coefficients from the ANCOVA regressions of AIs on age, and their corresponding standard errors.

Including results based on too few observations is likely to reduce reliability, therefore we chose to test with a cut-off of 15 observations per group and include assessments of fail-safe N’s for any significant finding (at a corrected alpha of *P* = 0.007). The method used was Rosenberg’s (Rosenberg 2005), which takes into account the weighted nature of the meta-analysis test, and its outcome can be interpreted as the number of studies averaging null-results which would be needed to render the observed *p*-value non-significant (*P* >= 0.007). Furthermore, effect heterogeneity was assessed by Cochran’s Q and the complementary Higgins’ I^2^, which both assess the contribution of dataset differences to the observed pooled effect. All tests were performed in R using the ‘metafor’ library (Viechtbauer 2010).

For the sex group comparisons, a 15-observation minimum threshold resulted in totals between 6867 and 6962 males versus 7708 to 7897 females, depending on the specific structure. For handedness, the totals were from 644 to 668 left handers versus 7298 to 7667 right handers. For meta-analyses of age-effects we applied the threshold of at least 15 observations per sex group and included an extra criterion based on the age-range of each dataset. Only results from datasets with a minimum 5-year range between their 1st and 3rd quartile (50 % of the dataset) were included.

To assess the pattern of statistically significant age effects across the lifespan, we performed a post-hoc weighted meta-regression of the age coefficients from each dataset on the corresponding median ages. Datasets were weighted by the square root of their corresponding sample size. The same criterion for dataset inclusion was used as described above.

### Population-level lateralization

*T*-scores and corresponding *P*-values were calculated for the difference between the mean AI and zero (i.e. the point of volumetric symmetry) for each structure and dataset, separately by sex. These were combined to assess population-level lateralizations for each structure, separately for each sex.

### Heritability of AIs

We estimated the heritability of volumetric asymmetries using the Genetics of Brain Structure (GOBS) dataset (McKay et al. 2014; Mitchell et al. 1996). This analysis included data from 1170 subjects of Mexican-American ancestry, belonging to 71 extended pedigrees. Heritability estimates were derived from variance-component analysis (Almasy and Blangero 1998). The method partitions the observed phenotypic variance into sub-components based on the relationship structures within the families, in order to estimate the proportion of overall phenotypic variance due to additive genetic effects. To calculate this family-based heritability estimate, the method requires large pedigrees and accurate kinship estimates between family members. For a more detailed description of the approach, applied to brain imaging measures, see (Chouinard-Decorte et al. 2014; Koran et al. 2014). These analyses were performed using SOLAR (Almasy and Blangero 1998) including age, sex, and ICV as covariates. For each of the seven structures we estimated the heritability of the AI and the heritability of the phenotypic correlation (i.e. genetic correlation) between left and right volumes. Lastly, we also assessed the phenotypic and genetic correlations across all seven AIs.

## Results

### AI heterogeneity between datasets

We observed notable heterogeneity in the AI distributions across datasets (Table 2 and Supplemental Information S6). Except for the hippocampus and putamen, dataset heterogeneity explained over 10 % (η^2^ > 0.1) of the total observed variance per structure. Likewise, heterogeneity attributable to different versions of FreeSurfer was also substantial, with η^2^ > 0.1 for AI’s of the nucleus accumbens, globus pallidus and thalamus.

**Table 2:** AI heterogeneity across datasets assessed by analysis of variance (ANOVA). The η^2^ statistic gives the proportion of the total variability attributed to mean AI differences between datasets or FreeSurfer versions. All mean AIs were significantly different from zero

Regions	Mean AI (σ^2^_within_)	N (observed)	η^2^ - dataset	η^2^ - FreeSurfer
Nucleus accumbens	-0.0072 (0.0061)	15,010	0.180	0.130
Amygdala	-0.0205 (0.0027)	15,167	0.103	0.017
Caudate nucleus	-0.0095 (0.0006)	15,105	0.279	0.014
Globus pallidus	0.0180 (0.0027)	14,932	0.171	0.142
Hippocampus	-0.0066 (0.0008)	15,046	0.070	0.010
Putamen	0.0194 (0.0008)	14,961	0.065	0.006
Thalamus	0.0211 (0.0009)	15,158	0.189	0.333

**Fig. 1:**
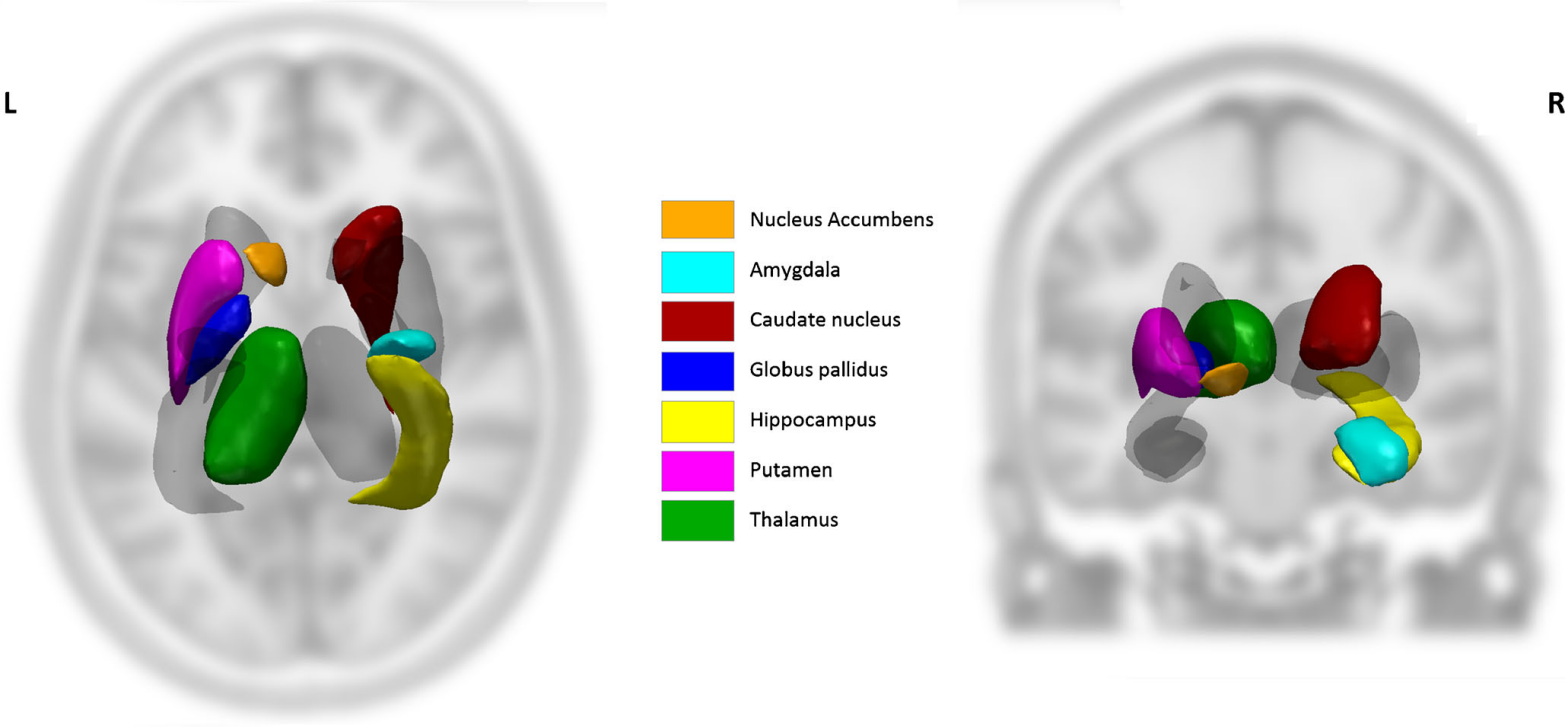
Visual representation of the 7 bilaterally paired structures, colored on the side of the relatively larger volume

Independent of dataset mean differences, the nucleus accumbens showed the most variable AI estimates, and the caudate nucleus was the least variable (see Table 2). The average variability around AI means, as a proportion of bilateral volume (σ^2^_within_*100), was 7.8 % for the nucleus accumbens and 2.5 % for the caudate nucleus. All structures showed highly significant mean lateralization, as well as consistency in mean direction of lateralization between the sexes (see Table 2 and Fig. 1, as well as Supplemental Information S7).

### Meta-analysis of group differences by sex and handedness

**Fig. 2:**
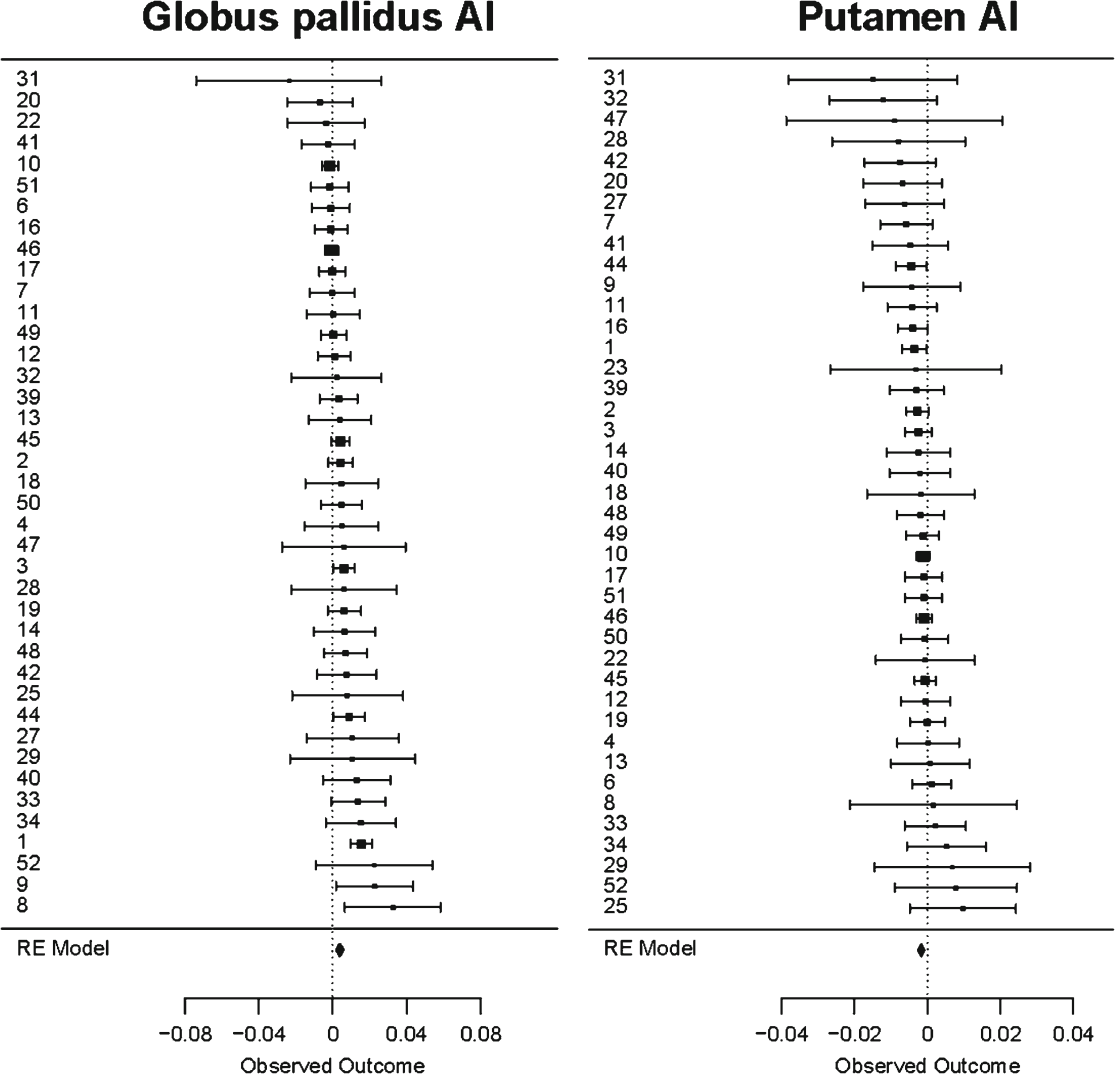
Forest plots of the mean sex differences in AIs per dataset, for the structures that showed significant sex effects in meta-analysis. For each structure, the datasets are ordered top-to-bottom by their estimated sex difference. The identities of the datasets are given by the numbers in the left-hand columns, with reference to Table 1. The size of a square is proportional to the weights assigned in meta-analysis. The confidence intervals are shown, as well as dashed vertical lines to indicate the point of no mean sex difference

After adjusting the significance threshold to *P* = 0.007 for multiple testing of seven structures, meta-analyses showed significant differences in AIs between males and females for the globus pallidus and putamen (Table 3 and Fig. 2), corrected for covariate effects of age and intracranial volume (ICV) within datasets. The direction of the sex difference for the putamen was negative (see Table 3), indicating a lower AI in males versus females, i.e. a rightwards shift in asymmetry in males. The opposite was found for the globus pallidus, where a leftward shift in AI was observed in males relative to females. Table 3 also reports the results of the study-heterogeneity and fail-safe N estimations. We observed no significant heterogeneity in sex effects across datasets for putamen and globus pallidus. A nominally significant sex effect (uncorrected *P* = 0.02) was also found for the hippocampus. Meta-analyses of handedness effects on AIs showed no significant group differences (uncorrected *P*-values > 0.1, results not shown).

**Table 3:** Meta-analyses results of (residualised) AI differences by sex, corrected for possible covariate effects of age and ICV. The significance threshold was Bonferroni-adjusted to 0.007 for the seven comparisons. Cochran’s Q and Higgins’ I^2^ are the statistics for the heterogeneity of effects. Highlighted in bold are the statistically significant results. Fail-safe N estimates are also given for the globus pallidus and putamen

Structure	Pooled effect	Standard error	*P*-value	N (datasets)	Cochran’s Q (*P*-value)	Higgins’ I^2^	Fail-safe N
Nucleus accumbens	0.002	0.002	0.34	14,652 (42)	76.9 (5.8*10^−4^)	51.2	0
Amygdala	5.7*10^−5^	7.4*10^−4^	0.94	14,859 (43)	33.37 (0.83)	0	0
Caudate nucleus	-1.3*10^−4^	6.5*10^−4^	0.84	14,723 (41)	75.13 (6.4*10^−4^)	50.78	0
Globus pallidus	0.004	0.001	2*10^−4^	14,575 (40)	52.49(0.073)	27.26	56
Hippocampus	0.001	4.5*10^−4^	0.02	14,765 (43)	19.99 (1.0)	0	0
Putamen	-0.002	4.1*10^−4^	4.5*10^−5^	14,604 (41)	24.49 (0.97)	0	53
Thalamus	-0.001	7.3*10^−4^	0.07	14,773 (41)	65.73 (0.006)	50.27	0

### Meta-analysis of age effects on AIs

**Fig. 3:**
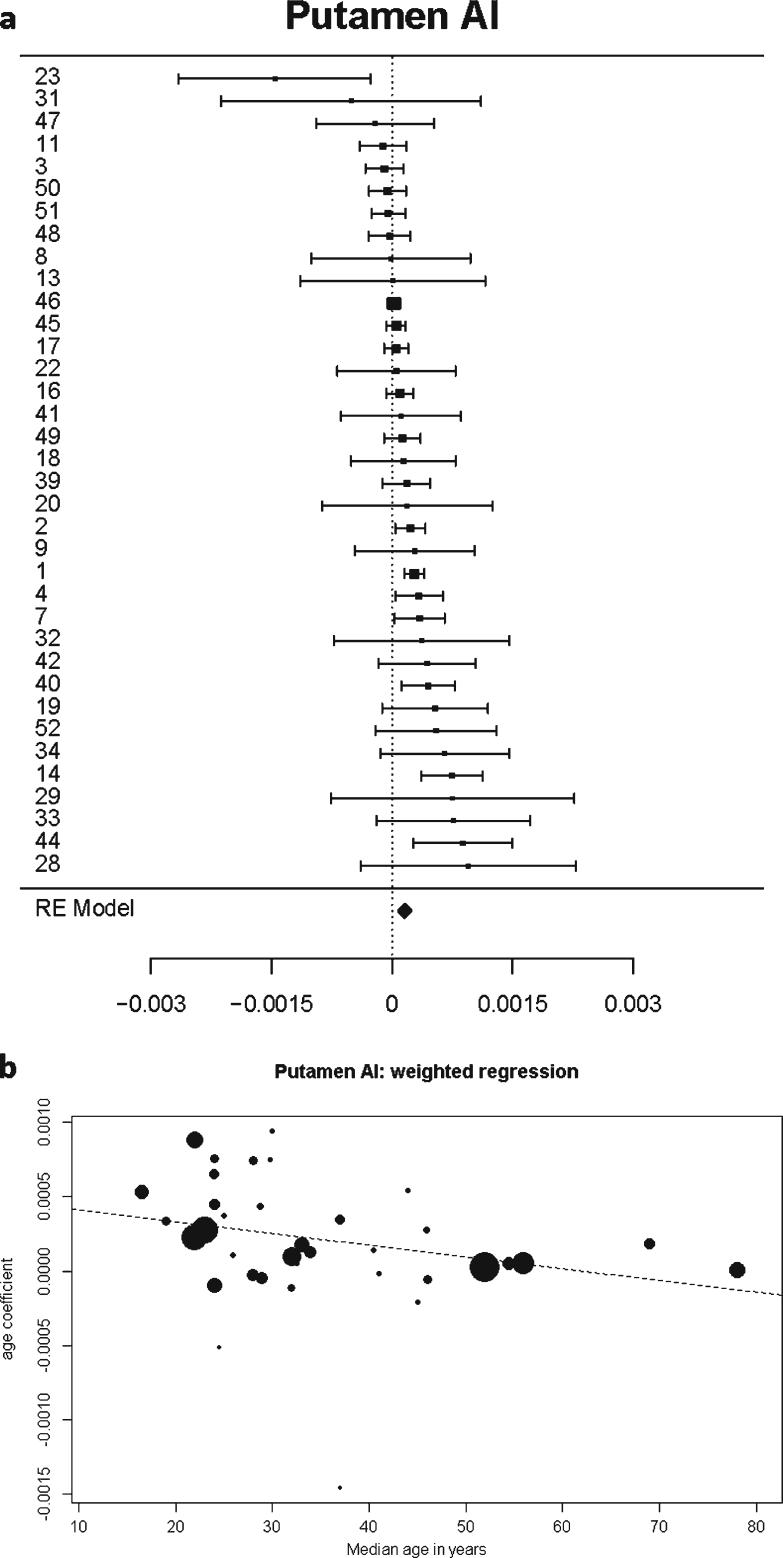
Results from meta-analysis of age effects. **a** Forest plot of the age coefficients for each dataset on putamen AI. The datasets are ordered top-to-bottom by their estimated age coefficient. The identities of the datasets are given by the numbers in the left-hand columns, with reference to Table 1. The size of a square is proportional to the weights assigned in meta-analysis. The confidence intervals are also depicted, as well as dashed vertical lines to indicate the point of an age coefficient with value zero. **b** Plot of the weighted regression of the age coefficients on each sample’s median age. The dotted line represents the best linear fit (*P* = 0.03). The size of a point is proportional to the square-root of a dataset’s sample size

After adjusting the significance threshold to *P* = 0.007 for multiple testing over seven structures, meta-analysis revealed a significant effect of age on the AI of the putamen (see Table 4 and Fig. 3a), corrected for covariate effects of sex and ICV within datasets. A positive pooled effect for the putamen indicated increasingly leftward shifts in asymmetry with increasing age. Table 4 also reports the results of the study-heterogeneity tests. While the heterogeneity tests for age effects on putamen AI were statistically significant, the estimate of a fail-safe N (*n* = 85) suggested that a biasing influence of study heterogeneity on the results was unlikely.

**Table 4:** Meta-analyses results for the age coefficients on AIs, corrected for sex and ICV. The significance threshold was Bonferroni-adjusted to 0.007 for the seven comparisons. Cochran’s Q and Higgins’ I^2^ are the statistics for the heterogeneity of effects. Fail-safe N estimates are also given for the putamen. The statistically significant results are highlighted in bold

Structure	Pooled effect	Standard error	*P*-value	Total N (datasets)	Cochran’s Q (*p*-value)	Higgins’ I^2^	Fail-safe N
Nucleus accumbens	2.1*10^−6^	2.1*10^−4^	0.99	12,073 (37)	229.16(5.8*10^−30^)	88.08	0
Amygdala	-1.8*10^−4^	6.9*10^−5^	0.009	12,287 (38)	74.75(2.3*10^−4^)	54.07	0
Caudate nucleus	5.9*10^−5^	5.0*10^−5^	0.24	12,150 (36)	148.03 (7.5*10^−16^)	78.14	0
Globus pallidus	-2.0*10^−4^	1.8*10^−4^	0.26	12,026 (35)	151.14 (1.0*10^−16^)	94.34	0
Hippocampus	-1.0*10^−4^	4.0*10^−5^	0.012	12,212 (38)	77.31(1.1*10^−4^)	48.69	0
Putamen	1.5*10^−4^	4.35*10^−5^	4.0*10^−4^	12,042 (36)	68.96 (5.3*10^−4^)	59.34	85
Thalamus	1.5*10^−4^	8.4*10^−5^	0.071	12,202 (36)	184.65 (3.0*10^−22^)	90.91	0

In our post-hoc analysis of age effects, by means of weighted meta-regressions, the putamen showed effects that changed across the median ages of our samples. We found larger effects of age in the younger datasets, compared to the older datasets (see Fig. 3).

### Heritability of AIs

AIs of the globus pallidus, hippocampus, putamen, and thalamus showed modest but statistically significant heritabilities, ranging from h^2^ = 0.15 to 0.27 (using a corrected alpha of *P* = 0.007; Table 5). For each subcortical region, we also estimated the genetic correlation (the proportion of variance that two traits share due to the additive effects of genes) between the absolute volumes of the left and right structures. While these correlations were all high (indicating partial pleiotropy), most were significantly different from 1 (i.e., complete pleiotropy; see Table 5). In other words, most genetic effects on volume variation are shared between the left and right hemispheres and therefore affect bilateral volumes of these structures, but some independent or quantitatively different genetic effects may operate uniquely on each hemisphere, thus constituting heritable effects on asymmetry. The nucleus accumbens also showed a suggestively significant heritability of its AI using an uncorrected alpha of 0.05 (see Table 5).

**Table 5:** Heritability estimates for the AIs, their corresponding standard errors and *P*-values, based on a large family dataset (GOBS). In the middle part of the table are the genetic correlations between left and right volumes (heritabilities of their phenotypic correlations), and test *P*-values for whether the genetic correlations differ significantly from 0 and 1. In the right-hand part of the table are the environmental and phenotypic correlation estimates between left and right volumes

Structure	AI heritability		Genetic correlation (ρ) between Left and Right			Phenotypic (ρ-phen) and environmental (ρ-env) correlation between Left and Right	
	h^2^ (se)	*P-value*	ρ (se)	P (ρ = 0)	P (ρ = 1)	ρ-phen	ρ-env
Nucleus accumbens	0.114 (0.06)	0.010	0.841 (0.07)	4*10^−10^	0.003	0.54	0.34
Amygdala	0.040 (0.05)	0.222	0.995 (0.03)	8*10^−24^	0.424	0.71	0.39
Caudate nucleus	0.096 (0.06)	0.053	0.974 (0.01)	2*10^−32^	0.021	0.85	0.56
Globus pallidus	0.148 (0.06)	0.002	0.823 (0.08)	8*10^−8^	0.005	0.57	0.45
Hippocampus	0.180 (0.06)	4*10^−4^	0.939 (0.02)	2*10^−25^	7*10^−4^	0.78	0.53
Putamen	0.270 (0.07)	8*10^−7^	0.899 (0.03)	5*10^−23^	4*10^−7^	0.78	0.58
Thalamus	0.228 (0.06)	2*10^−5^	0.824 (0.05)	1*10^−13^	4*10^−6^	0.68	0.56

Pairwise phenotypic and genetic correlations, assessed in the GOBS sample, are shown in Supplemental Information S8. Many of the phenotypic correlations were significant, but only the AIs of the putamen and thalamus showed a nominally significant genetic correlation (*r* = −0.48, uncorrected *P* = 0.037) in the presence of a significant phenotypic correlation (*r* = −0.26, *P* = 8.26*10^−23^). In other words, there may be genetic variability which affects these two AIs in opposite directions.

## Discussion

### Establishing effects of age, sex, and genetics

There is an inconsistent literature regarding basic biological factors that may affect subcortical and hippocampal asymmetries, including age, handedness, and sex. Subcortical asymmetries are subtle compared to some cerebral cortical asymmetries, and have so far only been assessed in small sample sizes, often with different analysis methods across studies (see Introduction). Compared to prior reports on subcortical asymmetries, our study analyzed a large number of datasets worldwide using a harmonized protocol and meta-analysis methods. To our knowledge, this was by far the largest ever study of healthy variation in any aspect of human brain asymmetry. The 52 datasets had technical and demographic differences, which appeared to influence the levels of asymmetry measured, but in this respect were representative of the heterogeneity that exists across cohorts worldwide. Given the scale of our study, and in contrast to literature-based meta-analyses, ours was not affected by publication bias nor by spurious results from underpowered studies. For future genome-wide screens, we also revealed significant heritabilities of asymmetries in a family sample.

We found reliable sex differences in asymmetries of the globus pallidus and putamen which, together with the hippocampus and thalamus, were also the most strongly heritable asymmetries among the seven structures analyzed. With increasing age, there were changes in the mean asymmetry of the putamen. Handedness was not detectably related to any subcortical asymmetry. The ENIGMA Consortium (Thompson et al. 2014) plans future genome-wide association studies in sample sizes comparable to, or greater than, that used here. Our data show which subcortical asymmetries are heritable and suitable for detecting subtle modulatory effects and group differences. Taken together, our heritability- and meta-analyses indicate that asymmetries of the putamen, globus pallidus, hippocampus and thalamus are the most likely structures through which genetic variation may impact lateralization for human cognition, its variability, and susceptibility to brain disorders.

From a developmental perspective, some human CNS lateralizations change throughout life (Kovalev et al. 2003). Asymmetries are detectable during fetal gestation behaviorally (Hepper 2013) and anatomically (Corballis 2013), so differential development between the two human brain hemispheres must, at least in part, be genetically coded in utero (Francks 2015). Three prior reports have suggested genetic contributions to variability in subcortical asymmetries based on twin-based heritability estimates. One found evidence for amygdala volumes being under strong genetic control, with higher heritability for the left than the right hemisphere (h^2^ = 0.80 and 0.55, respectively; (Hulshoff Pol et al. 2006)). Another found that genetic contributions to left and right volume variability were partly distinct for the nucleus accumbens and globus pallidus in particular (Eyler et al. 2014). A third found significant heritabilities of asymmetry indexes for the caudate nucleus and putamen, h2 = 0.17 and 0.32, respectively (Renteria 2013).

In terms of developmental biology and molecular genetics, the best studied model organism for CNS lateralization is the zebrafish. During the zebrafish’s development, there is a left-biased migration of a midline structure (the parapineal organ) that results in differential innervation of the bilateral epithalamus into the surrounding tissue, which later affects other brain regions (Concha et al. 2009). Specific molecular contributions to this process have been identified (Colombo et al. 2013). The relevance of this mechanism to humans is not clear, but a subcortical origin of lateralized development in the zebrafish brain suggests that similar or related mechanisms may be important in our species. Cerebral cortical lateralization may even be a downstream consequence of early subcortical lateralization.

For the putamen AI, asymmetry increased with age. In particular, the observed effect of age was more pronounced in samples with younger median ages, suggesting a non-linear relationship. Environmental or age-dependent genetic factors may contribute to this increased lateralization over time. To our knowledge, these associations have not been reported before, except for an opposite age effect for the putamen in 120 healthy, young adults (Abedelahi et al. 2013). We tested only linear effects of age at the dataset level, and these coefficients were meta-analyzed. Non-linear changes in AI with age might have gone undetected in our analysis, and may affect the measured linear effects. However, these meta-analyses were restricted to age effects observed in datasets with at least a 5-year age-range between the first and third quartile of participants. Most of our datasets had median ages between 20 and 60 years, so a linear regressor would have captured main effects of age on AIs, in these datasets, even if there were subtle non-linear effects. However, these factors should be considered when interpreting the pooled effect sizes reported.

Perhaps surprisingly, handedness had no detectable effect on subcortical asymmetries. However, as there are fewer left-handers than right-handers, the effective sample size was roughly one sixth for this analysis than for our analysis of sex differences. It remains possible, through even larger-scale meta-analysis, that handedness will relate to subcortical asymmetries. However, based on our present data, such effects must be very small.

### Dataset heterogeneity

Studies of subcortical structure have been greatly advanced by in vivo imaging. Even so, findings of population-level mean lateralizations of subcortical structures have been inconsistently reported. For example, there have been reports of the putamen being leftward lateralized on average (i.e. the left volume larger than the right (Giedd et al. 1996; Kang et al. 2015), as well as rightward lateralized (Abedelahi et al. 2013). Likewise the globus pallidus has been reported as leftward lateralized (Kang et al. 2015), as well as rightward lateralized (Wyciszkiewicz and Pawlak 2014). Similar discrepancies have also been found for the hippocampus (Kang et al. 2015; Niemann et al. 2000; Shi et al. 2009), amygdala (Makris et al. 2004; Niu et al. 2004; Szeszko et al. 1999) and the caudate nucleus (Abedelahi et al. 2013; Glenthoj et al. 2007; Raz et al. 1995; Vernaleken et al. 2007).

Here we used uniform image processing protocols, but our analysis showed substantial differences in mean AIs across datasets, which were partly attributable to different versions of FreeSurfer (see Table 2 and Supplemental Information S3). However, the majority of datasets (39 out of 52) were processed using version 5.3, so that our ability to assess the contributions of other FreeSurfer versions to AI variability was limited. Variability in image acquisition is likely a substantial source of dataset AI heterogeneity. The ability to distinguish different structures using MRI depends on the contrast achieved between different tissues. Subcortical structures and the surrounding tissue are often imperfectly contrasted, so that automated methods of image analysis must rely to some extent on atlas-derived information. These are often based on manual segmentations of existing datasets, which will reflect any mean asymmetries present in those datasets (Han and Fischl 2007; Patenaude et al. 2011). In addition, any subtle but uncorrected scanner magnetic field inhomogeneities may lead to geometric distortions in segmentation of brain structures (Han and Fischl 2007; Jovicich et al. 2009). These factors might bias segmentation, subtly affecting AI means. Manual segmentation does not avoid this problem, and can introduce asymmetric biases (Maltbie et al. 2012). In particular for assessing population variability (as opposed to as a diagnostic tool), automated methods clearly outperform manual segmentation in their reproducibility and feasibility for larger-scale studies (Guadalupe et al. 2014b; Morey et al. 2010).

In our study, all structures showed highly significant deviations from mean AI = 0, i.e. all showed population-level lateralization. Except for the hippocampus, the directions of significant mean AIs were in line with those reported in a study of 138 young adults, based also on subcortical volumes generated by FreeSurfer (Kang et al. 2015). However, given the caveats outlined above, we are cautious about interpreting the mean population AIs at face value. Different AI means across datasets may indicate which structures are more or less susceptible to methodological biases. The mean AIs for the hippocampus, amygdala, and putamen differed the least between datasets. The mean AI of the thalamus, on the other hand, showed the highest heterogeneity attributable to dataset heterogeneity (including FreeSurfer versions), and at the same time showed one of the strongest population-level AI lateralizations. This pattern is in line with our previous report that the hippocampus AI showed the highest scan-rescan correlation of all structures quantified with FreeSurfer (among the seven structures studied here), while the thalamus showed the second lowest scan-rescan correlation, in subjects scanned twice using varying protocols, and sometimes using different scanners with different field strengths (Guadalupe et al. 2014b).

In contrast to the substantial heterogeneity across datasets in mean AIs for some structures, there was less evidence for dataset heterogeneity in the effects of sex on mean AIs. We detected stable sex differences in AIs regardless of differences in age or ICV between and within datasets, and the sex differences were highly significant in our meta-analyses. The structures for which we detected sex differences in AIs showed L > R population-level asymmetry. For the globus pallidus this was more pronounced in males, while the opposite was observed for the putamen.

### Implications for future studies

Our study underlines the utility, and indeed the necessity, of analyzing subtle subcortical asymmetries in vast samples. Regarding clinical studies, some brain disorders may be associated with larger alterations in subcortical asymmetries than variables such as sex, handedness, and age. Nonetheless future studies linking subcortical asymmetries to disorders should be better powered if they analyze larger samples than used previously. Such studies will be possible within the ENIGMA Consortium.

It is reassuring that consistent sex differences could be measured in our study, even when AI means varied across cohorts. Some AIs were also heritable, based on studying relative-pair similarities. It is therefore clear that automated segmentation methods can measure meaningful individual differences in subcortical and hippocampal volumetric asymmetries (Guadalupe et al. 2014b; Hibar et al. 2015). It follows that genome-wide association studies of subcortical and hippocampal AIs are supported by this methodology, which will require very large samples for their success (Hibar et al. 2015; Stein et al. 2012).

## Electronic supplementary material

ESM 1 (PDF 262 kb)
