# Supplementary material for: Human subcortical brain asymmetries in 15,847 people worldwide reveal effects of age and sex
Source: Brain Imaging Behav. 2016 Oct 13;11(5):1497–514. doi: 10.1007/s11682-016-9629-z (PMC5540813; doi:10.1007/s11682-016-9629-z)
Supplement: Supplementary file 1 — (PDF 262 kb) [file 11682_2016_9629_MOESM1_ESM.pdf]

Supporting Information S1. Boxplots show the age distributions per cohort. The datasets are ordered vertically by median age, oldest at the top. On the *x*-axis are the age values (in years). The horizontal length of each box represents the age at the 2nd and 3rd quartile of their distribution (thus containing half the respective dataset). The vertical width of the boxes is proportional to the square root of the dataset sample size. Boxes are split at the median age, and the whiskers reach to the minimum and maximum ages.

Age distributions per dataset

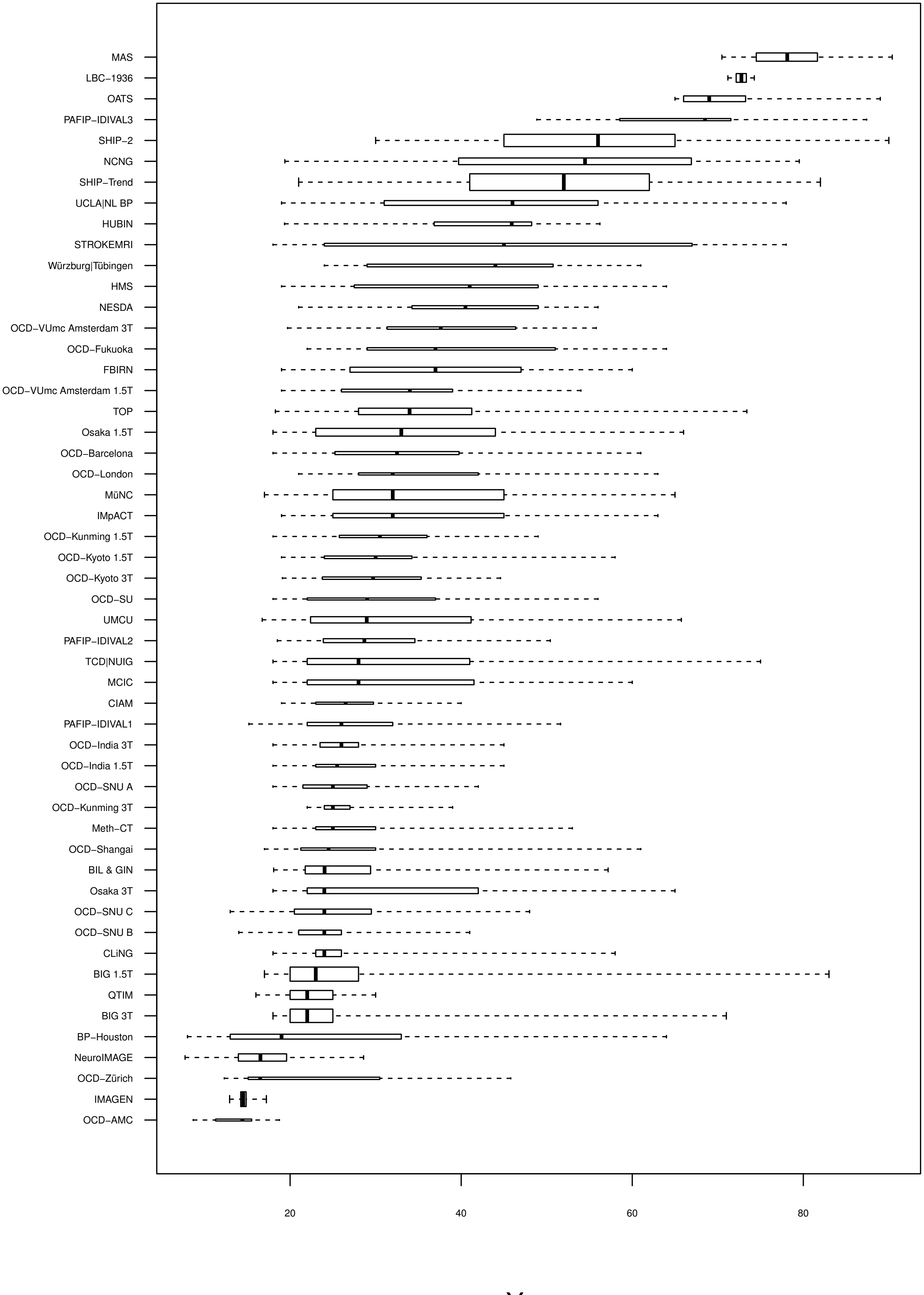

Supplementary Table S2: Dataset description and MR image acquisition details. On the left column are the reference numbers as used in Table I, Figures 2,3 and Supplementary Material S5.

Reference number	Dataset	Description	Scanner(s)	FreeSurfer version	T1 acquisition details
1	BIG 1.5T	The Brain Imaging Genetics database, split by scanner field strength. A population-based sample from Nijmegen, the Netherlands	1.5T (Sonata; Avanto) Siemens	5.3	MRI data in BIG were acquired with either a 1.5 Tesla Siemens Sonata or Avanto scanner or a 3 Tesla Siemens Trio or TimTrio scanner (Erlangen, Germany). Given that images were acquired during several smaller scale studies, the parameters used were slight variations of a standard T1-weighted three-dimensional magnetization prepared rapid gradient echo sequence (MPRAGE; 1.0×1.0×1.0 mm voxel size).
2	BIG 3T	The Brain Imaging Genetics database, split by scanner field strength. A population-based sample from Nijmegen, the Netherlands	3T (Trio; TimTrio) Siemens	5.3	MRI data in BIG were acquired with either a 1.5 Tesla Siemens Sonata or Avanto scanner or a 3 Tesla Siemens Trio or TimTrio scanner (Erlangen, Germany). Given that images were acquired during several smaller scale studies, the parameters used were slight variations of a standard T1-weighted three-dimensional magnetization prepared rapid gradient echo sequence (MPRAGE; 1.0×1.0×1.0 mm voxel size).
3	BIL & GIN	The Brain imaging of lateralization study at Groupe d'Imagerie Neurofonctionnelle, a sample of adult participants enriched in left-handers (45%), from Bordeaux, France (Mazoyer et al., 2015)	3T (Achieva) Philips	5.3	3D T1-weighted sequence: 3D-FFE-TFE; TR = 20ms; TE = 4.6ms; flip angle = 10°; inversion time = 800ms; turbo field echo factor = 65; sense factor = 2; matrix size = 256x256x180mm3; 1mm3 isotropic voxel size
4	BP-Houston	A subset of healthy controls from the Searching for Endophenotypes of Bipolar Disorders Study (BP)	a) 3T (Allegra) Siemens (1); b) 1.5 T (Gyroscan Intera) Philips & c) 1.5 T (Gyroscan Intera) Philips	5.3	a) T1-weighted MPRAGE scans: Flip angle = 8°, Echo time (TE)= 4.38ms, Repetition time (TR)= 1750ms, slice thickness = 1mm, matrix= 256x208, Number of slices =150.<br>b) T1-weighted spoiled gradient recalled (SPGR) scans: Flip angle = 40°, Echo time (TE)= 5ms, Repetition time (TR)= 24ms, slice thickness = 1mm, matrix= 256x256, Number of slices =150.<br>c) T1-weighted SENSE 3D scans: Flip angle = 6°, Echo time (TE)= 3.6ms, Repetition time (TR)= 8ms, slice thickness = 1mm, matrix= 256x256, Number of slices =150.
5	CIAM	A subset of healthy controls from Cortical Inhibition and Attentional Modulation: a study of psychosis (CIAM) - UCT.	3T (Allegra) Siemens	5.3	TR/TE, 2300/3.93 ms; flip angle, 12 degrees, FOV, 256 mm×240mm×160mm;andvoxelsize,1.3mm×1.0mm×1.0mm.
6	CLiNG	A sample of healthy controls from Clinical Neuroscience Göttingen	3 T (Trio Tim) Siemens	5.3	A T1-weighted, 3D magnetization prepared rapid gradient echo sequence (MPRAGE) (TR/TE/TI/FA=2250 ms/3.26 ms/900 ms/9°; image matrix = 256 x 256; duration 8 min and 26 sec) was acquired generating 192 sagittal slices with a voxel size of 1 mm3.
7	FBIRN	Healthy subjects from Function Biomedical Informatics Research Network (FBIRN)	a) 3T (TimTrio) Siemens & b) 3T (Discovery MR750) GE	5.1	a) MP-RAGE scan: scan plane=sagittal, TR/TE/TI=2300/2.94/1100ms, GRAPPA acceleration factor=2, flip angle=9°, resolution=256×256x160, FOV=220mm2, voxel size=0.86x0.86x1.2mm, and NEX=1.<br>b) IR-SPGR scan: scan plane=sagittal, TR/TE/TI=5.95/1.99/450ms, ASSET acceleration factor=2, a flip angle=12°, resolution=256×256x166, FOV=220mm2, voxel size=0.86x0.86x1.2mm, and NEX=1
8	HMS	Healthy subjects from the Homburg Multidiagnosis Study	1.5T (Sonata) Siemens	5.3	A T1-weighted, magnetization prepared rapid gradient echo sequence (MPRAGE) (TR/TE/TI/FA=1900 ms/4.0 ms/700 ms/15°; image matrix = 256 x 256) was acquired generating 176 consecutive sagittal slices with a voxel size of 1 mm3. ~5 min
9	HUBIN	A sample of healthy subjects from Human Brain Informatics	1.5T (Signa) GE	4.0	T1-weighted images, using a three-dimensional spoiled gradient recalled (SPGR) pulse sequence, were acquired with the following parameters; 1.5 mm coronal slices, no gap, 35° flip angle, repetition time (TR)= 24 ms, echo time (TE) = 6.0 ms, number of excitations (NEX) = 2, field of view (FOV) = 24 cm, acquisition matrix = 256 × 192.
10	IMAGEN	The Imaging-genetics consortium, an European international population-based sample (Schumann et al., 2010)	3T (Achieva) Philips; 3T Brucker; 3T (TrioTim) Siemens ; 3T (Verio) Siemens; 3T (Signa Excite) Brucker/GE & 3T (Signa HDx) GE	4.1	Magnetic resonance imaging data were acquired at 8 European centers, using a standardised 3 Tesla, T1-weighted gradient echo protocol (voxel size=1.1 mm isotropic) based on that from the ADNI initiative (http://adni.loni.usc.edu/methods/documents/mri-protocols/)
11	IMpACT	The International Multicentre persistent ADHD Genetics CollaboraTion: a subset Dutch healthy controls	1.5 T (Avanto) Siemens	5.3	All scans had a voxel size of 1×1×1 mm3, TR 2730 ms, TI=1000 ms, TE 2.95 ms, 176 sagittal slices, field of view 256 mm.
12	LBC-1936	Lothian birth cohort from 1936: Scotish sample of healthy older subjects (age~70) born on 1936, as part of a study on brain aging and cognition (Wardlaw et al., 2011)	1.5 T (Signa Horizon HDx) GE	4.3	3D IR-Prep T1-weighted whole brain FSPGR volumes were acquired in the coronal plane using a GE Signa Horizon HDxt 1.5 T clinical MRI scanner with manufacturer supplied eight-channel phased-array head coil; FOV = 256 x 256 mm, matrix = 192 x 192, 160 x 1.3 mm thick slices, 1 x 1 x 1.3 mm voxels, TR = 10 ms, TE = 4 ms and TI = 500 ms.
13	MAS	Sydney's Memory and Aging Study: an epidemiological sample of european subjects http://www.ncbi.nlm.nih.gov/pubmed/20637138	3T (Achieva Quasar Dual scanner) Phillips	5.3	3D T1-weighted structural (T1w TFE – turbo field echo) MRI, acquired coronally with repetition time TF= 6.39 ms, echo time TE = 2.9 ms, flip angle = 8°, matrix size = 256 × 256, field of view FOV = 256 × 256 × 190 mm3, and slice thickness = 1 mm with no gap between; yielding 1 × 1 × 1 mm3 isotropic voxels
14	MCIC	MIND Clinical Imaging Consortium formed by the Mental Illness and Neuroscience Discovery (MIND) Institute now the Mind Research Network (MRN; http//ww.mrn.org)	1.5T Siemens, 3T (Trio) Siemens & 3T (Signa) GE	4.0	TR = 2530 ms for 3 T, TR = 12 ms for 1.5 T; TE = 3.79 ms for 3 T, TE = 4.76 ms for 1.5 T; FA = 7 for 3 T, FA = 20 for 1.5 T; TI = 1100 for 3 T; Bandwidth = 181 for 3 T, Bandwidth = 110 for 1.5 T; 0.625×0.625 mm voxel size; slice thickness 1.5 mm; FOV 256×256×128 cm matrix
15	Meth-CT	Healthy controls from studies on methamphetamine use; University of Cape Town	3T (Magnetom Allegra) Siemens	5.3	T1-weighted, 3D-MEMPRAGE sequence: TR = 2530ms; graded TE = 1.53/2.21/4.89/6.57 m; flip angle = 7°; FOV = 256mm;slice thickness = 1mm
16	MüNC	The Münster Neuroimaging Cohort	3T (Gyroscan Intera), Philips	5.3	T1-weighted images were acquired using a fast gradient echo sequence (turbo field echo), repetition time 7.4 milliseconds, echo time 3.4 milliseconds, flip angle 9°, acquired over a field of view of 256 (feet-head [FH]) 204 (anterior-posterior [AP]) 160 (right-left) and reconstructed to cubic voxels of .5 mm .5 mm .5 mm [RL]) mm
17	NCNG	The Norwegian Cognitive NeuroGenetics sample : a population-based sample of European subjects (Espeseth et al., 2012)	1.5 T (Sonata) Siemens & 1.5 T (Avanto) Siemens	4.5	A Siemens Sonata 1.5 Tesla scanner (Siemens, Erlangen, Germany) with a conventional head coil was used. Two 3D MP-RAGE T1-weighted sequences (duration: 8 min 46 s) were run for all participants. Each volume consisted of 128 sagittal slices (1.33x1x1 mm3), with an in-plane voxel size of 1 mm3 (TR=2730 ms; TE=3.43 ms; TI=1000 ms; flip angle=7°; and 256×256 matrix). #2: A Siemens Avanto scanner was used to acquire two 3D MP-RAGE T1-weighted sequences (TR/TE/TI/FA=2400 ms/3.61 ms/1000 ms/8°; matrix=192×192; duration 7 min and 42 s per volume). Each volume consisted of 160 sagittal slices (1.25×1.25×1.20 mm3)
18	NESDA	A subset of healthy controls from the Netherlands Study of Depression and Anxiety.	3T (Achieva; Intera) Phillips	5.0	Imaging data were acquired at the Leiden University Medical Center, Amsterdam Medical Center, and University Medical Center Groningen, equipped with a SENSE-8 (Leiden University Medical Center and University Medical Center Groningen) or SENSE-6 (Amsterdam Medical Center) channel head coil. For each subject, anatomical images were obtained using a sagittal 3Dl gradient-echo T1-weighted sequence (TR=9 ms, TE=3.5 ms; matrix=256x256; voxel size=1x1x1mm3; 170 slices; duration=4.5 minutes).
19	NeuroIMAGE	A sample of european healthy controls and healthy siblings of ADHD patients	1.5 T (Sonata), Siemens and 1.5 T (Avanto), Siemens	5.3	1.5T MRI scanners were employed (Siemens SONATA and Siemens AVANTO; Siemens, Erlangen, Germany), using identical head coils (8-channel Phase Array Head Coil). T1-weighted whole-brain scan (MP-RAGE, 176 slices, acquisition matrix 256x256, voxelsize:1.0x1.0x1.0mm; TE/TR=2.95/2730ms, TI=1000ms, FA=7°; GRAPPA-acceleration 2)
20	OATS	One individual per family of The Older Australian Twins Study	1.5 T (Gyroscan) Philips; 3T (Achieva Quasar Dual) Philips; 1.5 T (Magnetom Avanto) Siemens; 1.5 T (Sonata) Siemens	5.3	(1) Sequence name T1TFE. In-plane resolution 1×1 mm. 256 × 256 matrix. Slice thickness 1.5 mm without gap. Number of slices: 150. Orientation: cor TR/TE: 7.73/3.7 ms Flip angle: 8°. Scan duration: 385 sec. (2) Acquisition parameters for T1-weighted structural MRI scans were: TR = 6.39 ms, TE = 2.9 ms, flip angle = 8°, matrix size = 256x256, FOV = 256x256x190, and slice thickness = 1 mm with no gap between; yielding 1x1x1 mm3 isotropic voxels (3) in-plane resolution= 1×1 mm, slice thickness= 1.5 mm, slice number=144, TR (Repetition time) =1530 ms, TE (Echo time) = 3.24 ms, TI (Inversion time)= 780 ms, flip angle=8 and NEX (Number of Excitations) =1. (4) in-plane resolution= 1×1 mm, slice thickness= 1.5 mm, slice number=144, TR (Repetition time) =1530 ms, TE (Echo time) = 3.24 ms, TI (Inversion time) = 780 ms, flip angle=8 and NEX (Number of Excitations) =1.
21	OCD-AMC	Control subjects from studies on OCD (pedriatric)	3T (Intera) Philips	5.3	3T (Philips Intera MR) matrix 256x256, 182 slices, voxel size 1x1x1.2mm
22	OCD-Barcelona	Control subjects from studies on OCD	1.5T (Signa Excite) GE	5.3	matrix 256x256, 130 slices, voxel size 1.2x1.2x1.2mm
23	OCD-Fukuoka	Control subjects from studies on OCD	3T (Achieva TX) Philips	5.3	3T (Philips Achieva TX) matrix 240x240, TR 8.2ms, TE 3.8ms, TI(inversion time) 240 ms, Flip angle 8 degree, FOV 240x240, NSA 1, Slice tickness 1mm, number of slice 190, voxel size 1.8x1.8.1.8mm, scan time 320s
24	OCD-India 1.5T	Healthy controls, examined using a structured interview to rule out a psychiatric diagnosis or neurological disease	1.5T (Vison) Siemens	5.3	1.5T (Siemens Vision): matrix 256x256, 160 slices, 0.98x0.98x 1mm
25	OCD-India 3T	Healthy controls, examined using a structured interview to rule out a psychiatric diagnosis or neurological disease	3T (Skyra) Siemens	5.3	3T (Siemens Skyra): matrix 256X256, 192 slices, voxel size 1.0 X 1.0 X 1.0 mm;
26	OCD-Kunming 1.5T	Control subjects from studies on OCD	1.5T (Signa Excite) GE	5.3	1.5T (Signa Excite) GE: matrix 256x256, 172 slices, voxel size 0.93x0.93x0.9mm
27	OCD-Kunming 3T	Control subjects from studies on OCD	3T (Achieva) Philips	5.3	3T (Achieva) Philips : matrix 228x228, 230 slices, FOV=250, voxel size 1.1x1.1x0.6mm
28	OCD-Kyoto 1.5T	Control subjects from studies on OCD	1.5 T (Gyroscan Intera) Philips	5.3	Three-dimensional volumetric acquisition of a T1-weighted gradient echo sequence produced a gapless series of contiguous, thin sagittal sections with the following parameters: flip angle, 15°; acquisition matrix, 256 × 256; field of view, 25 cm; section thickness, 1.5 mm; voxel size, 0.98 mm × 0.98 mm × 1.5 mm; TR, 9.9 ms; TE, 5.8 ms.
29	OCD-Kyoto 3T	Control subjects from studies on OCD	3T (Achieva 3.0 TX) Philips	5.3	The scanning parameters of the T1-weighted three-dimensional magnetization-prepared rapid gradient-echo (3D-MPRAGE) sequences were as follows: flip angle, 10 degrees; acquisition matrix, 256x256x170; field of view, 25.6 cm; section thickness, 1.0 mm; voxel size, 1.0 mmx1.0 mmx1.0 mm; TR, 7.1 ms; and TE, 3.3 ms.
30	OCD-London	Control subjects from studies on OCD	1.5 T (Signa) GE and 1.5T (Signa HDx) GE	5.3	Sequence 1: 3D SPGR, TR: 14.8ms, TE: 1.7ms, FA: 20º, Orientation: Axial, Matrix size: 256 x 256 x 124, Voxel size: 0.94 x 0.94 x 1.50; Sequence 2: 3D SPGR, TR: 10.8ms, TE: 5.0ms, FA: 18º, Orientation: Axial, Matrix size: 256 x 256 x 146, Voxel size: 1.09 x 1.09 x 1.10
31	OCD-Shanghai	Control subjects from studies on OCD	3T (Verio) Siemens	5.3	3T (Siemens verio) matrix 256x256, 192 slices, slice tickness 1.0mm, voxel size 1x1x1mm, TR 2300ms, TE 2.96ms, FOV 256x240, flip angle 9 degree
32	OCD-SNU A	Control subjects from studies on OCD	1.5T (Signa) GE	5.3	MPRAGE sequence were acquired in 176 contiguous axial slices: TR/TE = 1160/4.76 ms, field of view = 23 cm, flip angle = 15°, matrix 416x512, voxel size 0.45x0.45x0.90 mm.
33	OCD-SNU B	Control subjects from studies on OCD	1.5T (Avanto) Siemens	5.3	Contiguous 1.5-mm sagittal images were obtained with a three-dimensional T1-weighted spoiled gradient-echo sequence (echo time=5.5 ms; repetition time=14.4 ms; flip angle=20°; field of view=21×21 cm; matrix 256x256; voxel size 0.82x0.82x1.50 mm).
34	OCD-SNU C	Control subjects from studies on OCD	3T (Magnetom Trio) SIemens	5.3	High-resolution T1-weighted, three-dimensional MPRAGE (TR = 670 ms; TE = 1.89 ms; FOV = 50 mm; FA = 9°; matrix 256x256; voxel size 1.000x0.977x0.977mm).
35	OCD-SU	Control subjects from studies on OCD	3T (Magnetom Allegra) Siemens	5.3	T1-weighted MPRAGE sagittal 3D volume (TR=2300ms ;TE=3.93ms; TI=1100ms; 160 slices; FOV=256 x 240 mm; voxel size=1.3x1.0x1.0 mm3; slice thickness= 1 mm, flip angle=12 degrees
36	OCD-VUmc Amsterdam 1.5T	Control subjects from studies on OCD	1.5T (Sonata) Siemens	5.3	1.5T (Siemens Sonata) matrix 256x256, 160 slices, voxel size 1x1x1.5mm
37	OCD-VUmc Amsterdam 3T	Control subjects from studies on OCD	3T (Signa HDxt) GE	5.3	3T (Signa HDxt) matrix 256x256, 172 slices, voxel size 1x0.977x0.977 mm
38	OCD-Zurich	Healthy control subjects (adolescents and adults)	3T (Achieva) Philips	5.3	3T (Philips Achieva) matrix 240x240, 160 slices, voxel size isotropic 1x1x1mm, TR 8.14ms, TE 3.7ms, Flip angle 8 degree
39	Osaka 1.5T	Control subjects from the Japanese Osaka case-control studies of schizophrenia	1.5 T (Signa) GE & 3T (Signa HDxt) GE	5.3	T1-weighted IR-FSPGR sagittal 3D volume (TR=12.6 ms; TE=4.2 ms; TI=400 ms; 124 slices, matrix size=256x256, FOV=24.0x24.0 cm2, voxel size=0.9375x0.9375x1.4 mm3, slice thickness = 1.4 mm, flip angle=15°) T1-weighted IR-FSPGR sagittal 3D volume (TR=7.2 ms; TE=2.9 ms; TI=400 ms; 172 slices, matrix size=256x256x172, FOV=24.0x24.0 cm2, voxel size=0.9375x0.9375x1.0 mm3, slice thickness = 1.0 mm, flip angle=11°)
40	Osaka 3T	Control subjects from the Japanese Osaka case-control studies of schizophrenia	1.5 T (Signa) GE & 3T (Signa HDxt) GE	5.3	T1-weighted IR-FSPGR sagittal 3D volume (TR=12.6 ms; TE=4.2 ms; TI=400 ms; 124 slices, matrix size=256x256, FOV=24.0x24.0 cm2, voxel size=0.9375x0.9375x1.4 mm3, slice thickness = 1.4 mm, flip angle=15°) T1-weighted IR-FSPGR sagittal 3D volume (TR=7.2 ms; TE=2.9 ms; TI=400 ms; 172 slices, matrix size=256x256x172, FOV=24.0x24.0 cm2, voxel size=0.9375x0.9375x1.0 mm3, slice thickness = 1.0 mm, flip angle=11°)
41	PAFIP-IDIVAL1	Healthy controls from studies on schizofrenia	1.5T (Signa) GE	5.0	T1-weighted images, using a spoiled grass (SPGR) sequence, were acquired in the coronal plane with the following parameters: TE = 5ms, TR =24ms, NEX =2, FA=45o , FOV =26x19.5cm, slice thickness=1.5mm and a matrix of 256x192
42	PAFIP-IDIVAL2	Healthy controls from studies on schizofrenia	3T (Achieva) Phillips	5.3	Sagittal T1,TR=3000ms; TE=4.6ms; FA=8o; Voxel size=1x1x1 mm; Slice thickness=1mm ; Matrix size=321x312
43	PAFIP-IDIVAL3	Healthy controls from studies on schizofrenia	1.5T (Signa) GE	5.0	T1-weighted images, using a spoiled grass (SPGR) sequence, were acquired in the coronal plane with the following parameters: TE = 5ms, TR =24ms, NEX =2, FA=45o , FOV =26x19.5cm, slice thickness=1.5mm and a matrix of 256x192
44	QTIM	An aselect subset of twin-singletons of european descent from The Queensland Twin Imaging study.	4T (Bruker) Medspec	5.3	Scans were collected on a 4 Tesla Bruker Medspec scanner (Bruker, Germany). T1-weighted structural scans were acquired with acquisition parameters: TR=1500ms, TE=3.35ms, TI=700ms, flip angle=8°, 256 or 240 (coronal or sagittal) slices, FOV=240mm, acquisition voxel size 1.1x0.9x0.9mm.
45	SHIP-2	Population based sample from The Study of health in Pomerania (north-eastern Germany)	1.5 T (Avanto) Siemens	5.1	3D T1-weighted MRI sequence with the following parameters: MP-RAGE/ axial plane, TR=1900 ms, TE=3.4 ms and Flip angle=15° and an original resolution of 1.0 x 1.0 x 1.0mm3
46	SHIP-T	Population based sample from The Study of health in Pomerania (north-eastern Germany)	1.5 T (Avanto) Siemens	5.1	3D T1-weighted MRI sequence with the following parameters: MP-RAGE/ axial plane, TR=1900 ms, TE=3.4 ms and Flip angle=15° and an original resolution of 1.0 x 1.0 x 1.0mm3
47	STROKEMRI	Healthy controls from an ongoing stroke study, Oslo	3T (HDxT) GE	5.3	Sagittal T1-weighted FSPGR sequence (TE: 2.956, TR: 7.8 ms, flip angle: 12 degrees, voxel sixe=1 x 1 x 1.2 millimeter, number of slices: 166)
48	TCD|NUIG	Healthy subjects from TCD (Dublin) and NUIG (Galway)	TCD : 3T (Intera) Philips; NUIG : 1.5T (Magnetom), Siemens	TCD : 5.3 NUIG : 5.1	TCD: T1-weighted TFE gradient echo (TE (ms) 3.8 ; TR (ms) 8.4 ; Flip angle ( ) 8; FOV 230 ; Matrix 256 x 256; No. slices 180; slice thickness (mm) 0.9; Voxel-size (mm3) 0.9 x 0.9 x 0.9); NUIG: 1.5T: T1-weighted MP-RAGE (TE (ms) 4.38; TR (ms) 1140; Flip angle (°) 15; FOV 230 ; Matrix 512 x 512 (k-space interpolation from 256x256); No. slices 160; slice thickness (mm) 0.9; Voxel-size (mm3) 0.45 x 0.45 x 0.9)
49	TOP	Control subjects from Tematisk Område Psykoser (Thematically Organized Psychosis Research)	1.5T (Sonata) Siemens	5.3	Two sagittal T1-weighted magnetization prepared rapid gradient echo (MPRAGE) volumes were acquired with the Siemens tfl3d1_ns pulse sequence (TE = 3.93 ms, TR = 2730 ms, TI = 1000 ms, flip angle = 7°; FOV = 24 cm, voxel size= 1.33 x 0.94 x 1 mm3, number of partitions = 160)
50	UCLA|NL BP	Healthy controls from the Bipolar Genetics dataset. This NIMH-funded study is carried out at the University Medical Center Utrecht, the Netherlands, in a collaboration with the University of California Los Angeles	3T (Achieva) Philips	5.1	Three-dimensional T1-weighted images were acquired on a 3 Tesla Philips Achieva scanner (Philips Healthcare, Best, the Netherlands), equipped with an 8-channel SENSE-headcoil. Fast field echo scans with 200 contiguous sagittal slices (TE=4.6 ms, TR=10 ms, flip angle=8°, FOV=240 mm, 0.75 x 0.75 x 0.80 mm^3 voxels) were obtained.
51	UMCU	Healthy controls from two independent schizophrenia cohorts recruited at the University Medical Centre Utrecht, the Netherlands	1.5T (Achieva) Philips	5.1	A Three-Dimensional-Fast Field Echo (3D-FFE) on a 1.5T Philips Achieva scanner: TE=4.6ms, TR=30ms, flip angle=30°, FOV=256x256mm2) with 160-180 contiguous coronal 1.2 mm slices
52	Würzburg|Tübingen	Data set of healthy controls from a study on ADHD	1.5T (Avanto) Siemens	5.3	A high-resolution T1-weighted magnetization-prepared rapid gradient-echo imaging (MP-RAGE) 3D MRI sequence was obtained from each participant (TR: 2250ms, TE: 3.93ms, 8° flip angle, FOV: 256mm, matrix: 256×256, voxel size: 1×1×1mm3

Supplemental Information S3. List of datasets (arranged alphabetically) on which handedness analyses were performed, corresponding sample sizes and assessment methods.

Dataset	Left handed	Right handed	Assessment
**BIG 1.5T**	67	1205	Self-report
**BIG 3T**	56	1150	Self-report
**BIL & GIN**	205	248	Self-report
**CLiNG**	15	307	Self report confirmed by Edinburgh Handedness Inventory
**FBIRN**	5	173	Self-report
**HMS**	7	44	Self report confirmed by Edinburgh Handedness Inventory
**HUBIN**	6	90	Self-report
**IMAGEN**	160	1391	Self report confirmed by Purdue Pegboard test
**IMpACT**	15	126	Self-report
**LBC-1936**	34	522	Writing hand
**MCIC**	9	154	Annett Scale of Hand Preference
**MüNC**	14	729	Edinburgh Handedness Inventory: A threshold of 12 (out of 14) items was used to categorize as left- or right-handed.
**NCNG**	26	301	Self-report
**NESDA**	5	61	Self-report
**NeuroIMAGE**	45	333	Self-report
**OCD-VUmc Amsterdam 1.5T**	6	48	Self-report
**OCD-VUmc Amsterdam 3T**	7	31	Self-report
**Osaka 1.5T**	28	409	Self report confirmed by Edinburgh Handedness Inventory
**Osaka 3T**	11	226	Self report confirmed by Edinburgh Handedness Inventory
**SHIP-2**	57	1053	Self-report
**SHIP-Trend**	97	1943	Self-report
**STROKEMRI**	6	46	Self-report
**TOP**	22	279	Self-report
**UCLA|NL BP**	20	140	Self-report
**UMCU**	36	227	Self-report

Supplemental Information S4 : Left-right flip checks

Of special importance was to assure the correct correspondence between the left/right orientation of the processed image data and the original subject space. In contrast to the other axes (antero-posterior or superior-inferior), the correct orientation on the left-right axis is not directly identifiable from visual features, making it difficult to readily detect any erroneous image flips during processing. Such problems are much more unlikely since the adoption of the nifti imaging standard (http://nifti.nimh.nih.gov/), but they can still be a potential source of artifact if the raw (often DICOM-formatted) data is processed with incorrect assumptions (SPM documentation, p. 157; http://www.fil.ion.ucl.ac.uk/spm/doc/manual.pdf).

Because the ENIGMA protocol starts after the raw (often DICOM-formatted) data has been converted into an imaging standard (or converted by FreeSurfer itself), this meant that conversion from the DICOM format was the most likely step where any error could have taken place. This was assessed using several strategies, depending on the available information at each site. The **BIL & GIN**, **FBIRN**, **MAS**, **NESDA** and **OATS** samples had made use of paramagnetic fiducial markers on a subset of their subjects, thus eliminating orientation ambiguity. In **QTIM** and **SHIP**, subjects with a known unilateral brain abnormality were used to check the correct orientation of the image after conversion. In **BIG**, **CLiNG**, **HMS** and **OCD-SU,** a few examples were manually checked for mismatches between the DICOM and nifti header information, i.e. a correct flip from 'radiological' to 'neurological' orientation. Finally, we checked the consistency between several, commonly used, DICOM to nifti conversion tools and DICOM images generated from different manufacturers/models (using examples downloaded from the manufacturer's websites). The convertors used in this step were: "mri_convert" (https://surfer.nmr.mgh.harvard.edu/pub/docs/html/mri_convert.help.xml.html), " MRIConvert" (http://lcni.uoregon.edu/downloads/mriconvert), "dcm2nii" (http://www.cabiatl.com/mricro/mricron/dcm2nii.html) and "spm_dicom_convert" (http://www.fil.ion.ucl.ac.uk/spm/).

Given that these checks yielded no problems, and that the datasets where no error was detected comprised 60% of the total meta-analysis sample, we were confident that such orientation errors must have been very unlikely.

Supplementary Information S5.

Plots of the relationship between ICV and subcortical AIs in the BIG 3T sample. Regression analyses of linear and quadratic relationships revealed only linear effects of ICV with AIs of the nucleus accumbens, caudate nucleus and putamen (as indicated by the dashed lines).

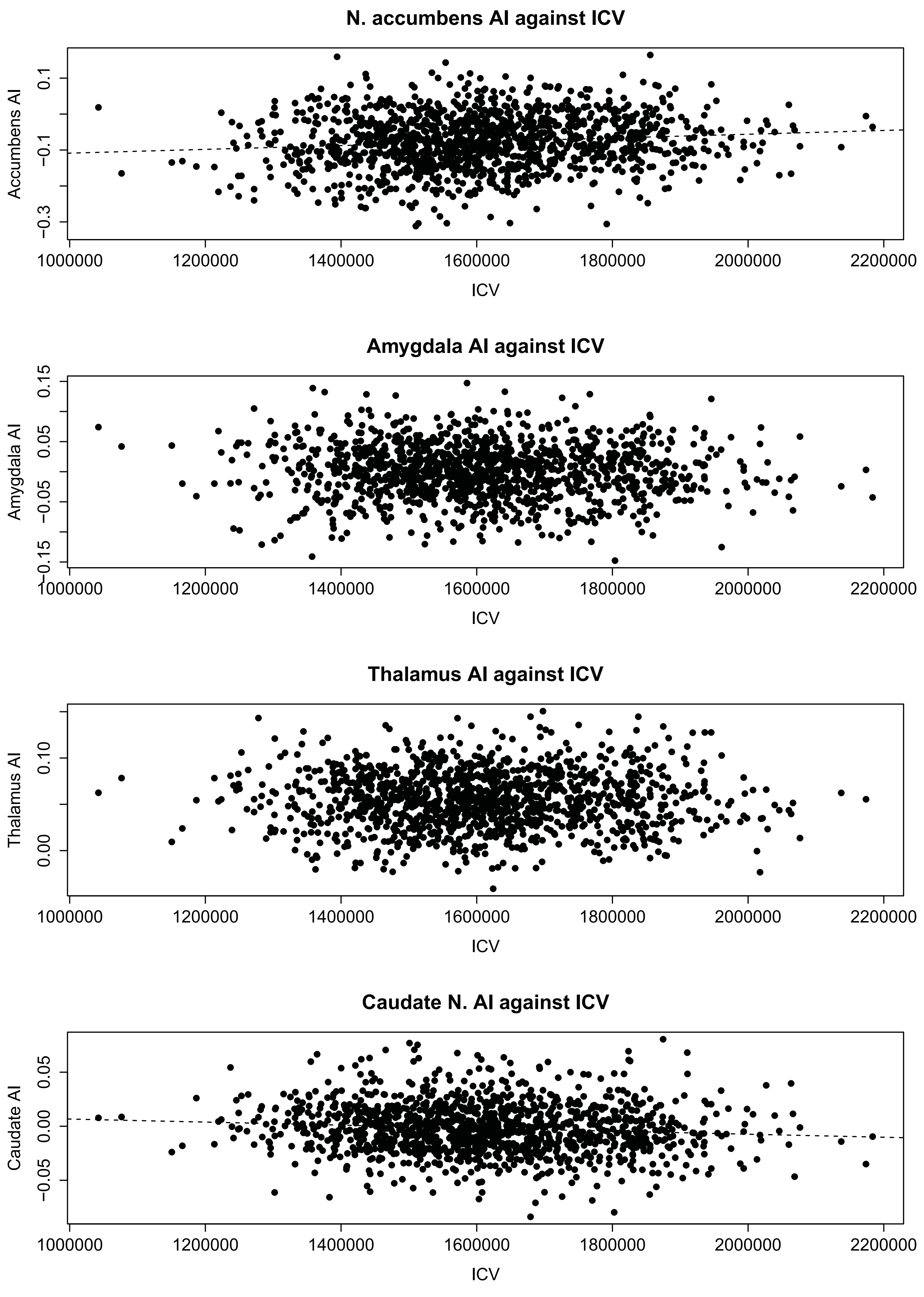
N. accumbens AI against ICV
Accumbens AI
0.1
−0.1
−0.3
ICV
1000000
1200000
1400000
1600000
1800000
2000000
2200000
Amygdala AI against ICV
Amygdala AI
0.15
0.05
−0.05
−0.15
ICV
1000000
1200000
1400000
1600000
1800000
2000000
2200000
Thalamus AI against ICV
Thalamus AI
0.10
0.00
ICV
1000000
1200000
1400000
1600000
1800000
2000000
2200000
Caudate N. AI against ICV
Caudate AI
0.05
0.00
−0.05
ICV
1000000
1200000
1400000
1600000
1800000
2000000
2200000

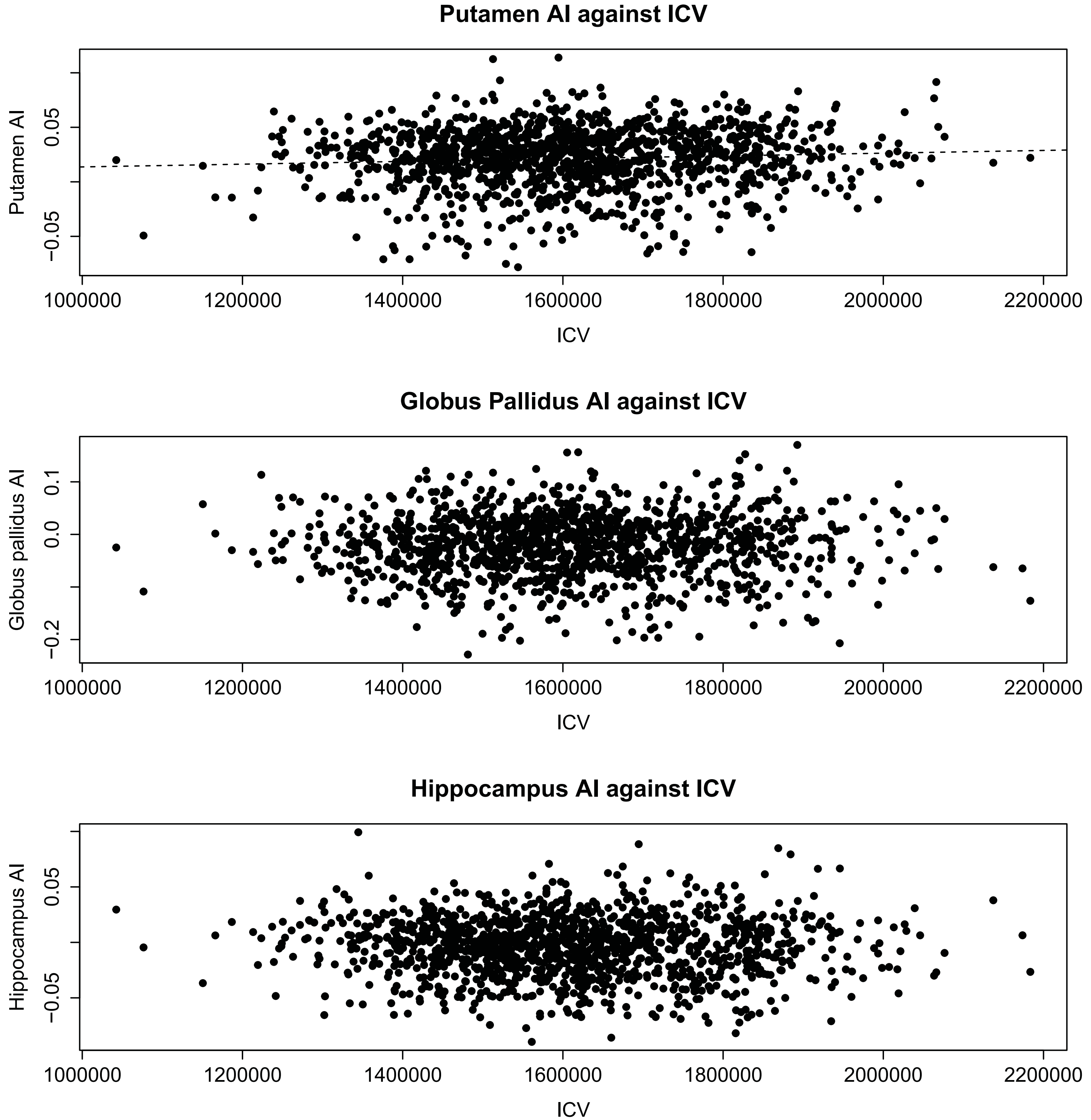
Putamen AI against ICV
Putamen AI
0.05
−0.05
1000000
1200000
1400000
1600000
1800000
2000000
2200000
ICV
Globus Pallidus AI against ICV
Globus pallidus AI
0.1
0.0
−0.2
1000000
1200000
1400000
1600000
1800000
2000000
2200000
ICV
Hippocampus AI against ICV
Hippocampus AI
0.05
−0.05
1000000
1200000
1400000
1600000
1800000
2000000
2200000
ICV

Supplementary Information S6.

Boxplots of AI distributions for each dataset and structure. For each structure, the datasets are ordered top-to-bottom by their median AIs. The identities of the datasets are given by the numbers in the lefthand columns, with reference to Table 1. The horizontal length of each box represents the 2nd to 3rd quartile of the AI distribution (i.e. containing half of subjects in each dataset) and split at the median AI. The vertical width of each dataset's box is proportional to the square root of its sample size. The whiskers show the minimum and maximum values (curtailed in cases where the outer box boundary was reached). The vertical dotted lines indicate the points of perfect symmetry, AI=0.

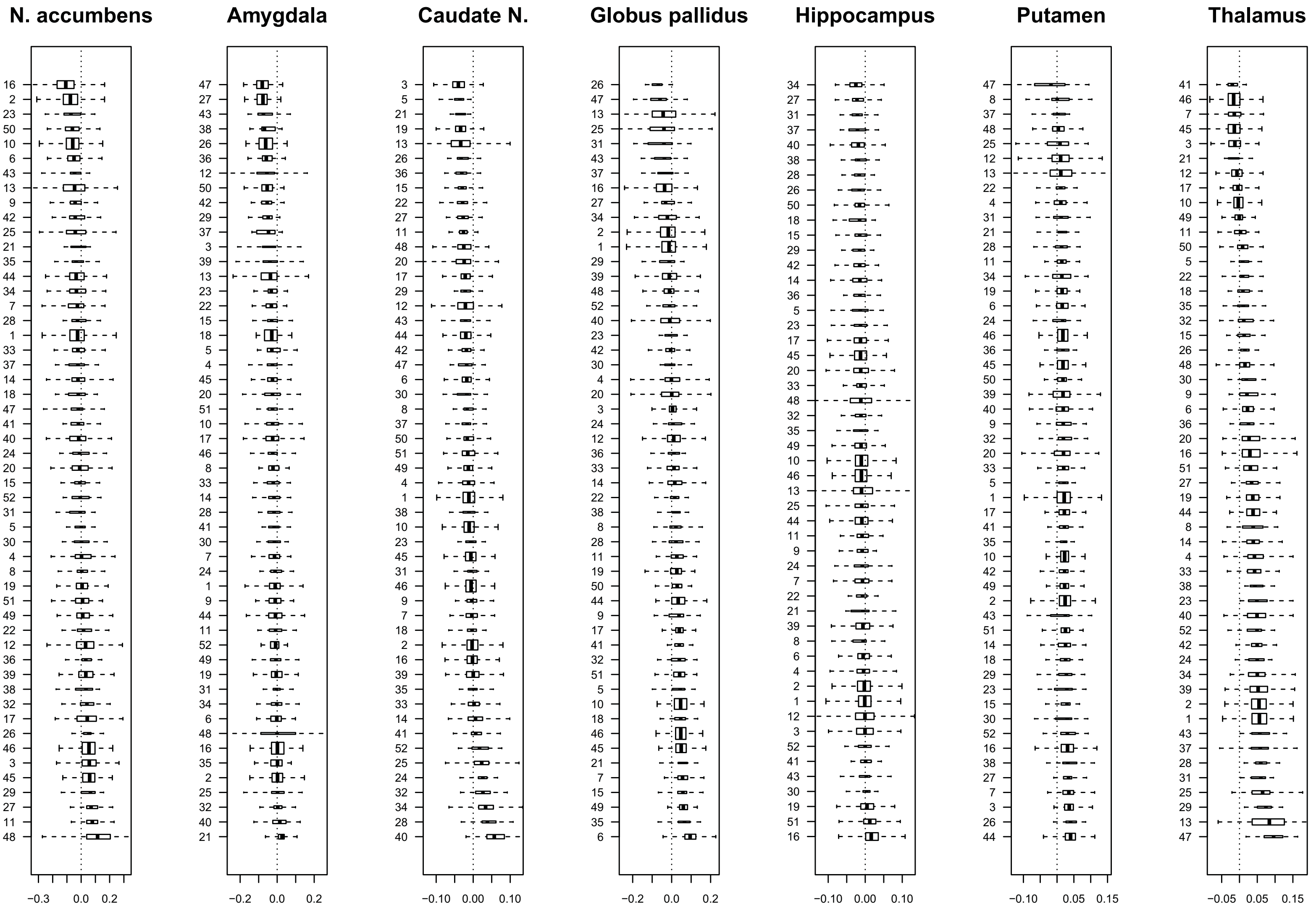

N. accumbens
Amygdala
Caudate N.
Globus pallidus
Hippocampus
Putamen
Thalamus
-0.3 0.0 0.2
-0.2 0.0 0.2
-0.10 0.00 0.10
-0.2 0.0 0.2
-0.10 0.00 0.10
-0.10 0.05 0.15
-0.05 0.05 0.15

Supporting Information S7. Meta-analyzed results from testing population-level lateralization (mean AI's ≠ 0) separately by sex. A positive Z-score indicates leftward asymmetry in volume (L>R), while a negative Z-score reflects a rightward asymmetry (R>L).

Females	N	z-score
Nucleus accumbens	7957	-11.01
Amygdala	8049	-33.36
Caudate nucleus	7980	-34.92
Globus pallidus	7892	23.61
Hippocampus	7971	-22.67
Putamen	7920	59.86
Thalamus	8043	41.43

Males	N	z-score
Nucleus accumbens	7053	-4.80
Amygdala	7118	-32.72
Caudate nucleus	7125	-31.12
Globus pallidus	7040	31.16
Hippocampus	7075	-20.14
Putamen	7041	53.16
Thalamus	7115	33.44

**Phenotype 1**	**Phenotype 2**	**Phenotype correlation**	**Phenotype correlation P**	**Genetic correlation**	**Genetic correlation P**
AI_amygdala	AI_accumbens	0.00	0.90028	-0.99	0.0126487
AI_amygdala	AI_caudate	0.10	0.0001192	0.85	0.0914741
AI_amygdala	AI_hippocampus	0.00	0.9357421	-0.81	0.0356209
AI_amygdala	AI_pallidum	0.03	0.2340173	-0.42	0.3471581
AI_amygdala	AI_putamen	0.09	0.0005557	0.13	0.716055
AI_amygdala	AI_thalamus	-0.06	0.0209367	-0.10	0.7943838
AI_caudate	AI_accumbens	-0.08	0.0040859	0.32	0.3575671
AI_caudate	AI_hippocampus	-0.03	0.3293978	-0.36	0.2211863
AI_caudate	AI_pallidum	0.05	0.0393021	-0.11	0.752416
AI_caudate	AI_putamen	0.12	4.46E-06	-0.14	0.6170061
AI_caudate	AI_thalamus	0.04	0.1032485	0.31	0.3141842
AI_hippocampus	AI_accumbens	0.00	0.9842037	-0.11	0.6521278
AI_hippocampus	AI_pallidum	0.02	0.4847126	0.10	0.6775807
AI_hippocampus	AI_putamen	0.01	0.7405954	0.04	0.8433021
AI_hippocampus	AI_thalamus	0.11	3.29E-05	0.10	0.6507085
AI_pallidum	AI_accumbens	0.07	0.0068105	0.18	0.5735795
AI_pallidum	AI_putamen	0.05	0.0446912	-0.12	0.6430572
AI_pallidum	AI_thalamus	-0.21	7.16E-16	-0.26	0.3783415
AI_putamen	AI_accumbens	-0.02	0.4199311	-0.61	0.0202141
AI_putamen	AI_thalamus	-0.26	8.26E-23	-0.48	0.0365587
AI_thalamus	AI_accumbens	-0.05	0.0873988	-0.14	0.6142865

Supplementary Information S8. Phenotypic and genetic correlations among AIs in the GOBS dataset.
